# Glucose-Powered Ultrasmall Chemotactic Nanorobots for Retinal Degeneration Treatment

**DOI:** 10.1021/jacs.5c15651

**Published:** 2025-11-21

**Authors:** Xiaohui Ju, Kateřina Palacká, Roshan Velluvakandy, Jan Michalička, Martin Pumera

**Affiliations:** † Future Energy and Innovation Laboratory, Central European Institute of Technology, 48274 Brno University of Technology, Purkyňova 123, 61200 Brno, Czech Republic; ‡ Department of Toxicology and Molecular Epidemiology, 145206 Institute of Experimental Medicine of the Czech Academy of Sciences, Vídeňská 1083, 14220 Prague, Czech Republic; § Faculty of Science, Charles University, Albertov 2038, 12800 Prague, Czech Republic; ∥ Central European Institute of Technology, 48274 Brno University of Technology, Purkyňova 123, 61200 Brno, Czech Republic; ⊥ Advanced Nanorobots & Multiscale Robotics Laboratory, Faculty of Electrical Engineering and Computer Science, VSB - Technical University of Ostrava, 17. listopadu 2172/15, 70800 Ostrava, Czech Republic; # Department of Medical Research, China Medical University Hospital, China Medical University, No. 91 Hsueh-Shih Road, Taichung 40402, Taiwan; ▲ Department of Chemical and Biomolecular Engineering, Yonsei University, 50 Yonsei-ro, Seodaemun-gu, Seoul 03722, Korea

## Abstract

Retinal degeneration poses a growing global health challenge with limited effective treatments. Current options, such as intravitreal injections of therapeutic drugs, are severely constrained by the vitreous humor barrier, a dense, gel-like matrix that limits drug diffusion to the retina. Micro/nanorobots with active propulsion have emerged as promising platforms for targeted drug delivery to overcome biological barriers. Here, we report the design of chemotactic nanorobots that can actively overcome the vitreous humor to target the retina. Single-atom engineering is utilized to construct ultrasmall nanorobots that catalytically convert endogenous glucose into mechanical propulsion, enabling active navigation through the vitreous barrier toward retinal tissues. Both ex vivo tissue and in vivo mouse models confirm the nanorobots’ ability to overcome vitreous viscosity and target retinal cells due to their ultrasmall sizes (less than 10 nm) and active motion. In a mouse model of induced retinal degeneration, these nanorobots exert potent dual antioxidant and immunomodulatory activities, markedly delaying disease progression. Mechanistic studies at the gene expression level further elucidated the molecular basis of these therapeutic effects. These promising findings highlight the potential of single-atom engineered chemotactic nanorobots as effective nanomedicine, paving the way for their application as active drug delivery platforms in noninvasive treatment of ocular diseases.

## Graphical abstract

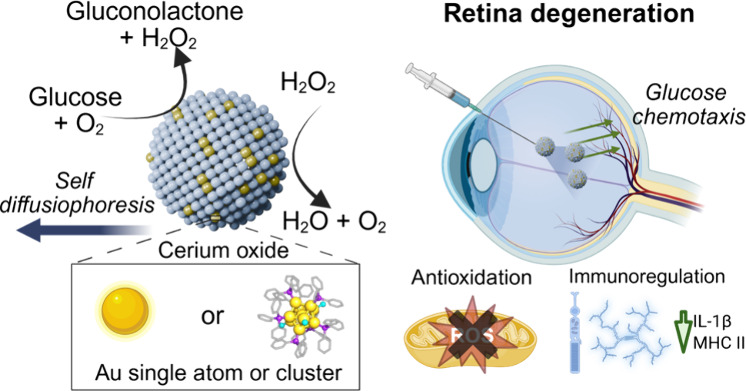

## Introduction

Retinal degenerative diseases, including age-related macular degeneration (AMD) and diabetic retinopathy, affect hundreds of millions worldwide, with AMD projected to affect 288 million people by 2040.^1^ Clinical management of AMD typically involves repeated intravitreal injections of antivascular endothelial growth factor (anti-VEGF) agents for the wet form, and oral supplementation with antioxidant vitamins and minerals for the dry form. Although these interventions can slow disease progression and preserve vision, they are associated with significant limitations, including the need for repeated injections, risk of ocular complications, and variable patient response. The pathogenesis of retinal degeneration is multifactorial, with inflammation and oxidative stress playing a significant role. Aging and environmental factors contribute to the accumulation of metabolic byproducts and reactive oxygen species (ROS), which can trigger oxidative damage and structural remodeling in retinal tissues.^2^ Recent advances have revealed another unexpected aspect, showing that inflammation-induced overactivation of immune cells, including microglia, significantly contributes to retinal degeneration.^3^ Immunity in the retina acts like a double-edged sword: while essential for maintaining health and function, its dysregulation due to a compromised immune-privileged status accelerates degenerative diseases.^4,5^ Overactive microglia cells release pro-inflammatory cytokines, causing further damage to photoreceptors and the retinal pigment epithelium. These findings add new dimensions to understanding and addressing retinal degeneration.

Effective treatment of retinal degeneration remains hindered by the challenge of efficient drug delivery to the retina. The vitreous humor—a dense, gel-like extracellular matrix—acts as a physical and biochemical barrier that significantly restricts the diffusion and distribution of drugs administered via intravitreal injection. This barrier limits therapeutic concentrations reaching target retinal cells and contributes to variable and often suboptimal treatment outcomes. Therefore, overcoming the vitreous barrier and enhancing drug penetration to retinal tissues are critical steps toward improving treatment efficacy and long-term disease management. Nanorobotics has the potential to revolutionize pharmacological and surgical treatments^6^ by overcoming the anatomical and physiological barriers that restrict access to nanomedicine to the retina. A decade has passed since Peer Fischer’s group first demonstrated the use of magnetic helical microswimmers to navigate through viscous, heterogeneous fluids.^7^ In 2018, the same group showcased how these magnetic micropropellers could traverse the vitreous humor to reach the retina.^8^ In the past five years, only a few studies have explored microrobots for targeted drug delivery in the eye, limited either to light propulsion^9,10^ or magnetic maneuvering.^11,12^ None of these studies has evaluated the practical application and potential damage to the surrounding tissues of these nanorobots in vivo. Moreover, the use of externally driven micro/nanorobots propelled by magnetic fields and visible light presents challenges in the development of clinical setups.

The development of chemically propelled nanorobots that use biocompatible fuels, such as glucose from biological fluids, to generate motion and perform tasks, presents exciting opportunities for advancing biomedical interventions.^13,14^ Current glucose-based nanorobotic systems suffer from relatively large size due to enzyme incorporation,^15^ or high concentration of metal component, causing potential cytotoxicity.^16^ In this work, we propose the design of a single-atom engineered ultrasmall nanorobotic system with chemotactic behavior toward glucose to overcome the viscous vitreous humor and reach the retina. Atomically anchored gold atoms or gold clusters can catalyze glucose oxidation to generate motion. These single atoms and clusters are anchored onto fully biocompatible ultrasmall-sized colloidal cerium oxide nanoparticles, which have already been proven as therapeutic agents for ocular treatments.^17,18^ We have previously shown that nanorobots with asymmetrical gold distribution display an enhanced motion and chemotactic behavior in the presence of glucose, with propulsion resulting from neutral self-diffusiophoresis during glucose decomposition.^19^ The concept of nanoarchitectonics provides a framework for designing ultrasmall nanorobots through the controlled assembly of atomic- and molecular-level components.^20,21^ This approach enables precise functionalization and tunable interactions with biological environments, which are essential for effective ocular delivery. Here, we use both ex vivo tissue and in vivo mouse models to comprehensively demonstrate that glucose-powered nanorobots can overcome vitreous humor barriers to reach the retina. Moreover, the enhanced accumulation of the nanorobots in the retina did not cause any adverse effects on retinal function but rather exhibited therapeutic effects. Gene level expression reveals that this is due to their dual function of antioxidation and immunological regulation to slow down retinal damage, as illustrated in Figure 1. The biocompatibility and adaptability of these nanorobots underscore their potential as versatile drug delivery platforms capable of transporting therapeutic agents to the retina via multiple modalities. These findings lay the groundwork for translational applications and highlight the underexplored potential of nanorobots in overcoming biological barriers for ocular drug delivery.

**Figure 1:**
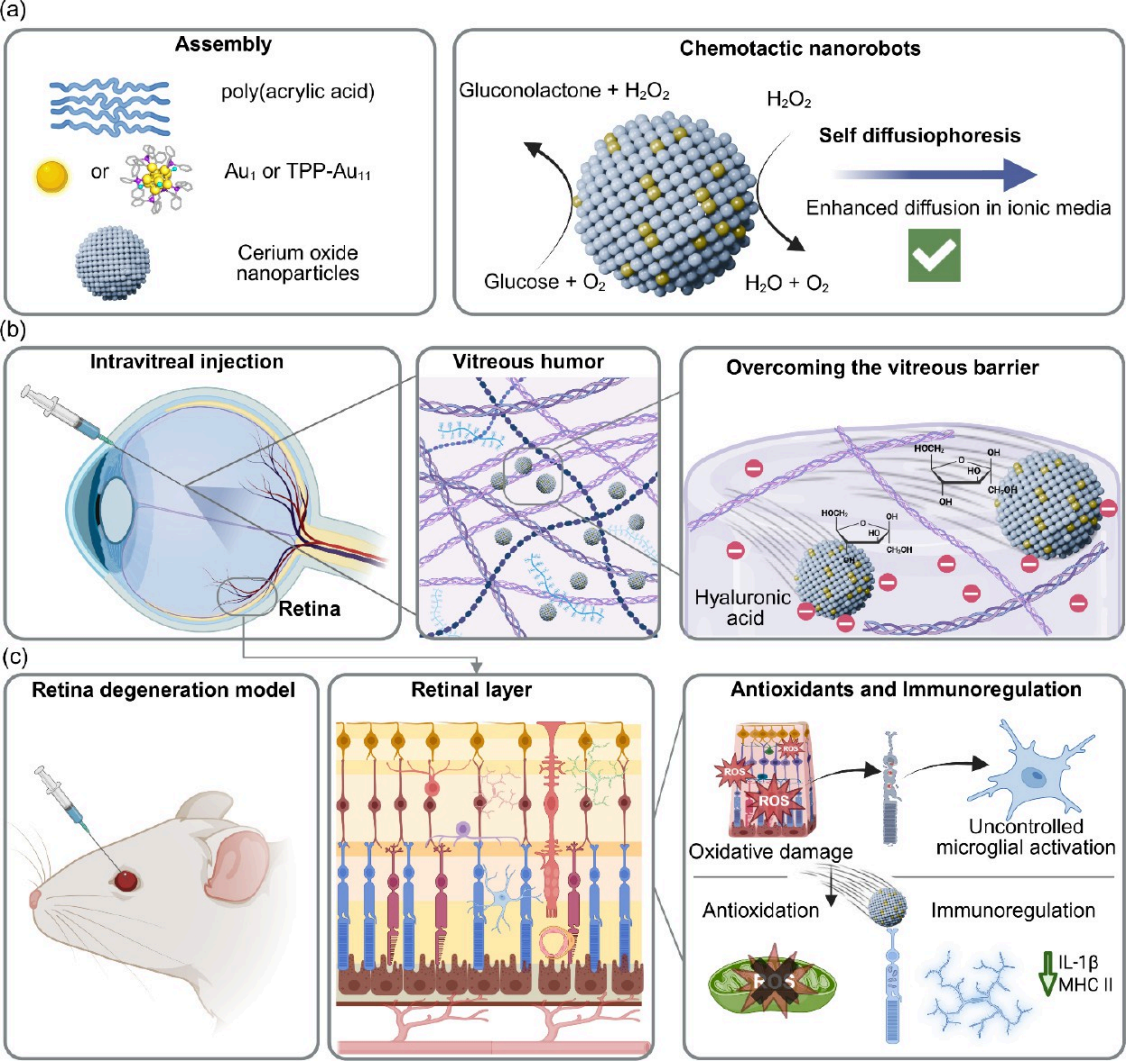
Schematic representation of glucose-powered ultrasmall gold nanorobots as potential drug delivery platforms for retina degeneration. (a) Assembly of glucose-powered nanorobots and its main catalytic-based propulsion generated through diffusiophoresis, including a schematic of the chemotactic nanorobot and its reactive sites. Left: Au site (gold) catalyzes the conversion of glucose to gluconolactone and hydrogen peroxide. Right: cerium oxide site (gray) catalyzes the decomposition of hydrogen peroxide to water and oxygen. TPP-Au_11_ nanoclusters Au_11_(PPh_3_)_7_Cl_3_ structural representation is reproduced from ref 22 by Truttmann et al. Available under a CC-BY 4.0 license. Copyright 2022 Wiley. (b) The assembled glucose-powered nanorobots can overcome complex media such as vitreous humor and possess enhanced diffusion. (c) In an in vivo experimental mouse model of retinal degeneration, through intravitreal injection, the glucose-powered nanorobots can reach the retina. These biocompatible nanorobots also exhibit potential therapeutic effects to fight against retinal degeneration through antioxidant and immunomodulatory effects. The scheme is created with BioRender.com.

## Results and Discussion

### Fabrication and Characterization of Gold Cluster-Decorated Cerium Oxide Nanorobots

The fabrication of the retina-targeting nanorobots consists of three steps: the preparation of ligand-protected Au nanoclusters, the fabrication of poly­(acrylic acid)-coated cerium oxide nanoparticles, and the assembly of these two parts (Figure 1a). The triphenylphosphine-stabilized undecagold nanocluster, Au_11_(PPh_3_)_7_Cl_3_ (TPP-Au_11_), belonging to the “magic number series,” has been extensively studied for heterogeneous catalysis, particularly oxidations, due to its precise atomic structure, superior catalytic efficiency, and high stability compared to gold nanoparticles and single atoms.^22^ “Magic number” gold clusters are exceptionally stable nanostructures, where free electrons are confined within a spherically symmetric potential well of the metal core. Their stability arises when the electron count aligns with the “magic” number series (2, 8, 18, 20, 34, 58,...), as described by the superatom model, with their size and stability further influenced by the selection of surface ligands.^23^ The TPP-Au_11_ clusters were synthesized and purified following previously described methods.^24^ Characterization of the unsupported clusters was performed by UV–visible spectroscopy (UV–vis), attenuated total reflection Fourier transform infrared spectroscopy (ATR-FTIR), and X-ray photoelectron spectroscopy (XPS), confirming the undecagold structural arrangement with protective TPP ligands (Figure S1). To characterize the synthesized Au clusters on the atomic scale, high-resolution, high-angle annular dark-field imaging in scanning transmission electron microscopy mode (HAADF-STEM) was performed. The TPP-Au_11_ clusters dispersed on a carbon membrane after synthesis (Figure S2) exhibited an average diameter of 0.79 ± 0.08 nm, consistent with a reported value of 0.8 nm.^25^ Poly­(acrylic acid)-functionalized ultrasmall cerium oxide nanoparticles (CeNPs, core size ∼3 nm, hydrodynamic diameter ∼6 nm) were synthesized according to our previous work and served as the support for immobilizing the gold clusters.^26,27^ The nanorobots (TPP-Au_11_–CeNPs) were assembled by wet impregnation of TPP-Au_11_ clusters with PAA-coated CeNPs, yielding a gold loading of 0.7 at %. We then compare the TPP-Au_11_–CeNPs with gold single atoms anchored on PAA-coated CeNPs (Au_1_–CeNPs) to provide insights into the catalytic efficiency and chemotactic behavior as glucose-powered nanorobots.^19^ We utilize both NPs to evaluate the catalytic activity of Au species in glucose oxidation. While Au_11_ clusters exhibit similar functionality to Au_1_, they offer the added advantage of potentially serving as more stable catalytic units, resisting aggregation. TPP ligands surrounding Au_11_ clusters have been extensively used for mitochondria targeting and provide future potential to be tuned for suborganelle targeting.^28^

We monitored the hydrodynamic diameters, surface charges, band gaps, crystallite structures, and chemical oxidation states of CeNPs, Au_1_–CeNPs, and TPP-Au_11_–CeNPs using UV–vis, XPS, dynamic light scattering (DLS), and X-ray diffraction (XRD) (Figure 2a). Confirming the morphological assembly of TPP-Au_11_ clusters adsorbed onto CeNPs is possible using high-resolution HAADF-STEM. Figure 2b presents an example of suspected TPP-Au_11_ clusters decorating the CeNPs. Background contrast variation around the CeNPs is visible, potentially caused by the PAA polymer coating. Enlarged areas marked with green circles in Figure 2b highlight two suspected Au clusters, identified based on their distinctive morphology and size. Additional representative images of suspected Au clusters are provided in Figure S3. It is appropriate to note that STEM imaging poses several challenges. First, there is a lack of a strong Z-contrast between ^58^Ce and ^79^Au atoms, which creates large NPs or small clusters, respectively, and thus rather a thickness contrast dominates in the HAADF images. Second, the electron beam can induce knock-on damage to the small Au clusters, which has an immediate effect on their structure during STEM imaging and thus hinders the observation of the clusters in their original form. To provide better evidence of the structural appearance of Au clusters among CeNPs, we have prepared a mixture with ∼1000× higher concentration of Au_11_ clusters and examined it with HAADF-STEM (Figure S4).

**Figure 2:**
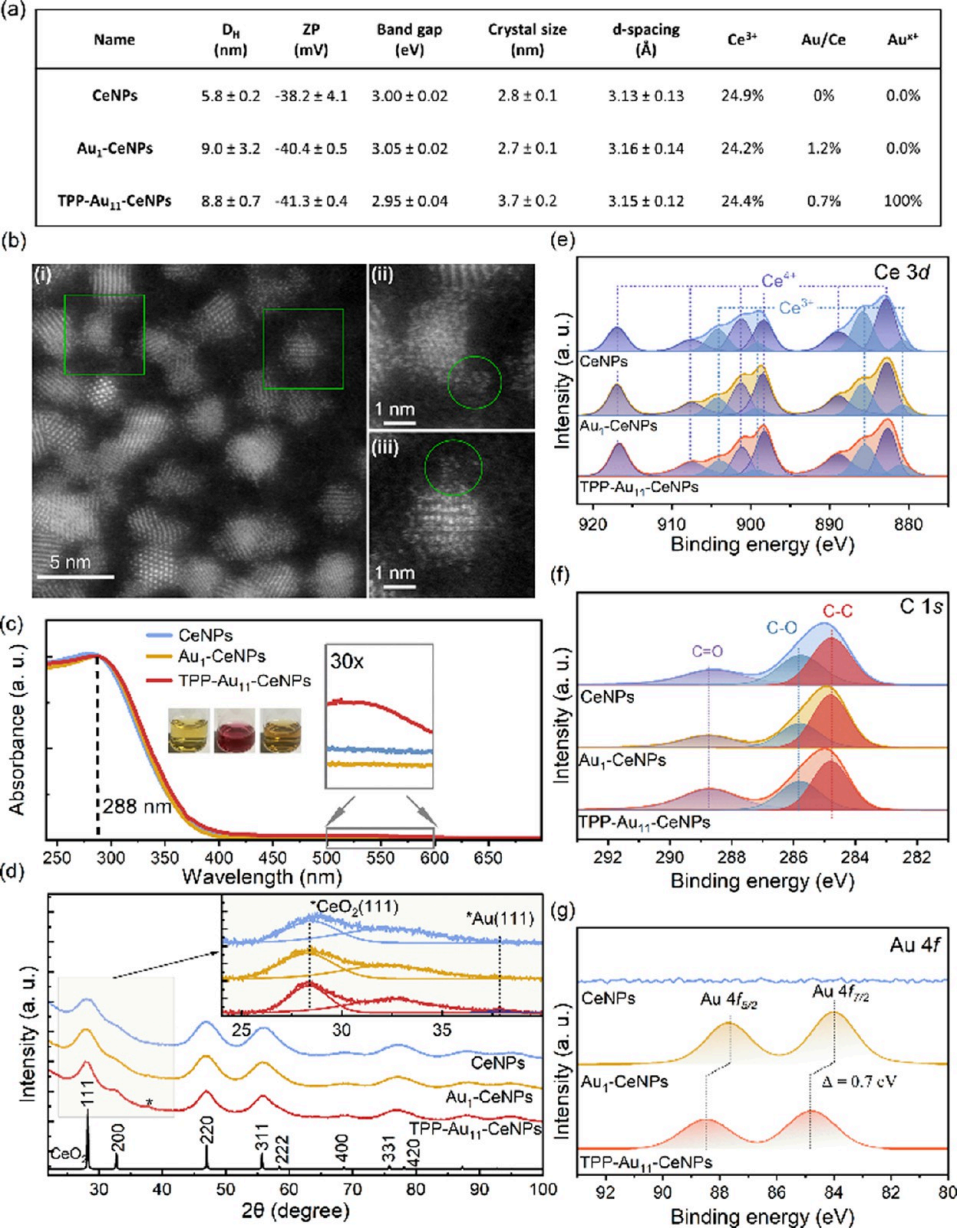
Physiochemical characterization of synthesized samples. (a) Table of summarized physicochemical properties of PAA-CeNPs (hereafter referred to as CeNPs), single atom decorated PAA-CeNPs (Au_1_–CeNPs), and TPP-Au_11_ cluster decorated PAA-CeNPs (TPP-Au_11_–CeNPs), including hydrodynamic diameter (*D*_H_ based on number) and zeta potential (ZP) measured by DLS; bandgap measured by UV–vis; crystal size and *d*-spacing of the (111) plane measured by XRD; Ce^3+^/Ce percentage, Au/Ce atomic percentage, and Au^*x*+^/Au percentage measured by XPS. (b) Morphological characterization of Au-based nanorobots by HAADF-STEM. (i) HAADF-STEM image of TPP-Au_11_–CeNPs. Green rectangles highlight regions enlarged in (ii) and (iii). (ii) Enlarged view of a selected region of TPP-Au_11_–CeNPs, with a green circle indicating a visible TPP-Au_11_ cluster. (iii) Another enlarged region of TPP-Au_11_–CeNPs showcases a visible TPP-Au_11_ cluster. (c) UV–vis absorbance spectra of three samples normalized based on the CeNPs absorbance peak at 288 nm. Pictures show vials of 20 mg L^–1^ of sample colloidal dispersions in water. Inserts show the detailed broadening from 500 to 600 nm region with 30× magnification. (d) XRD pattern of the measured sample compared with the CeO_2_ fluorite structure (bottom, black); *d*-spacing of (111) was analyzed in the enlarged graph. (e) Core-level Ce 3d spectra of measured samples fitted with 5 doublets. The fitting of the Ce^3+^ and Ce^4+^ peaks follows the parameters proposed by Lykhach et al.^31^ The Ce^3+^/Ce percentage was calculated from the integrated areas of the assigned peaks and is listed in (a). (f) Core-level C 1s spectra of measured samples fitted with 3 peaks, attributed to the poly­(acrylic acid) coating onto the CeNPs. (g) Core level spectra of Au 4f*.*

TPP conjugation shifts the zeta potential of TPP-Au_11_–CeNPs to more positive values compared to CeNPs and Au_1_–CeNPs. The PAA coating is confirmed by core level C 1s spectra (Figure 2f), which indicates the nanoparticles’ potential to resist protein corona formation, and enhanced biostability as confirmed in our previous study.^26,27^ All three samples display a similar cerium oxide fluorite structure with a crystallite size of approximately 3 nm and a bandgap of about 3 eV, indicating no lattice expansion from the accommodation of gold single atoms and nanoclusters on the oxide support (Figure 2a–d). The core level Au 4f spectra of Au_11_ cluster-decorated CeNPs show a slight upshift of the Au 4f_7/2_ peak compared to the Au metallic form at 84.0 eV (Figure 2g), attributed to reduced final state screening due to decreased metallic conductivity and cluster charging when the photohole is not promptly neutralized by the support material.^29^ Such a higher shift toward positive charge is also reflected by the increase in reduced Ce^3+^ percentage of TPP-Au_11_–CeNPs at 28% compared with ∼25% for the other two samples, indicating a strong metal–support interaction (SMSI).^30^

### Glucose-Enhanced Nanorobot Diffusion and Chemotaxis

Gold-based nanoparticles have been reported to possess glucose oxidation catalytic activities mimicking the glucose oxidase (GO_*x*_) enzymes.^32^ One of the glucose oxidation reaction products is hydrogen peroxide (H_2_O_2_).^33^ Hydrogen peroxide can undergo catalytic disproportionation in the presence of cerium oxide nanoparticles, which mimic the activity of catalase by facilitating the conversion of H_2_O_2_ to water and oxygen. Thus, gold-anchored cerium oxide nanoparticles utilize the advantages of single-atom and cluster engineering to couple two cascade reactions into one single and simple confinement. Figure 3a shows that such coupled reaction kinetics can be monitored by measuring the UV absorbance of NPs in the presence of glucose by calculating the *A*_400_/*A*_290_. Colloidal CeNPs have a characteristic peak around 290 nm in relation to their concentration, while when CeNPs react with H_2_O_2_, a stable intermediate called cerium peroxo/hydroperoxo complex forms, giving rise to a differential spectrum with a peak at 400 nm.^34^ In the presence of glucose, Au_1_–CeNPs and TPP-Au_11_–CeNPs both can catalyze the coupled reaction, reaching a plateau after 2 min, while CeNPs remained inert in the presence of glucose. Au_1_–CeNPs exhibited a slightly higher catalytic efficiency compared to TPP-Au_11_–CeNPs (Figure 3b). Due to the asymmetrical distribution of the single atoms and gold nanoclusters analogous to the mechanism of Janus structure, these nanomotors can generate sufficient chemical gradients leading to self-diffusiophoresis. Both types of particles exhibited enhanced diffusion coefficient constants in the presence of glucose (Figure 3c). At physiological glucose levels (∼1 mg mL^–1^), Au-decorated nanomotors exhibit a 2-fold increase in swimming speed, indicative of active Brownian motion (Figure 3d).

**Figure 3:**
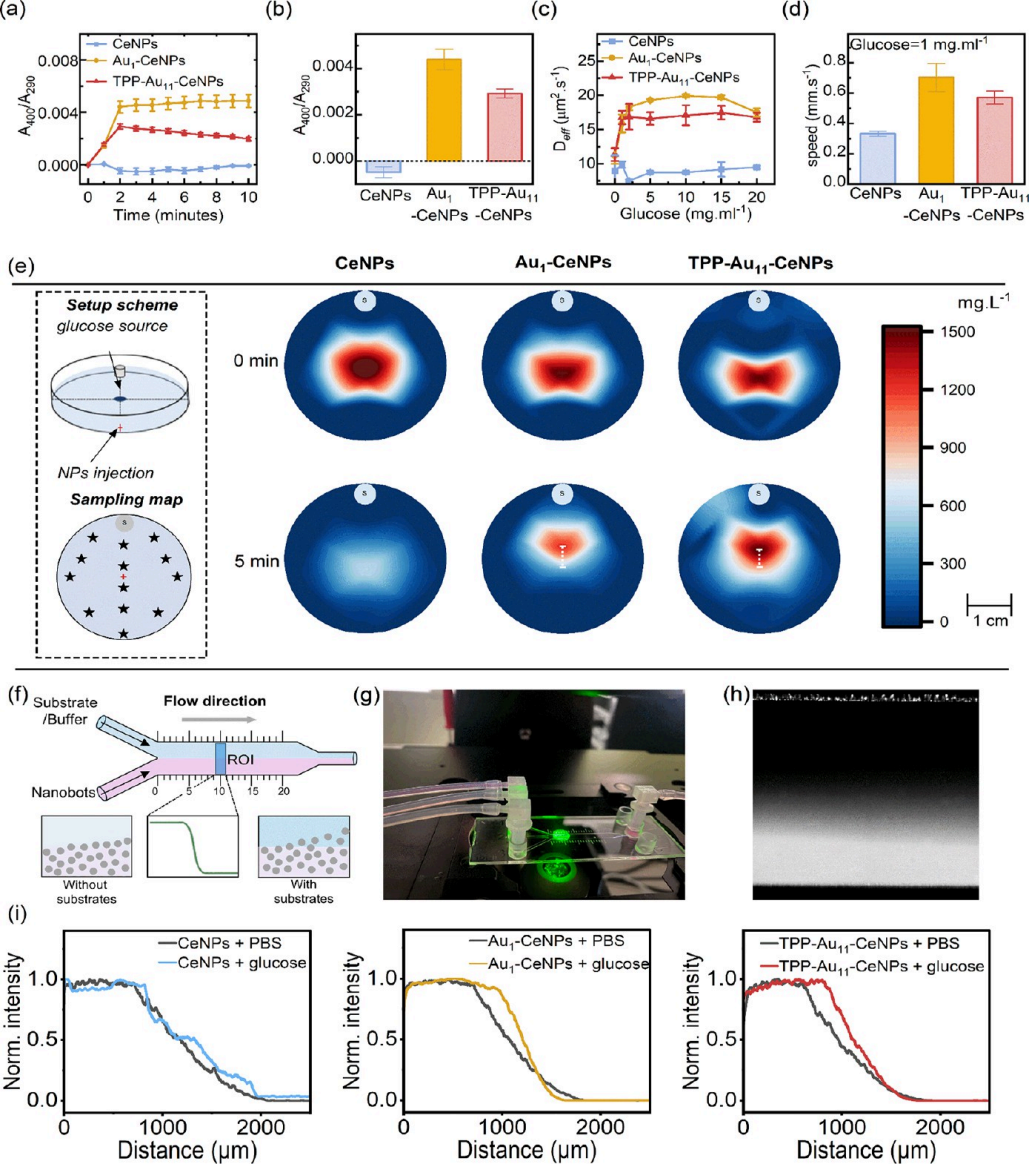
Glucose-mediated enhanced diffusion of nanorobots and their chemotaxis behavior. (a) Glucose oxidase- and catalase-mimicking activities coupled with reaction kinetics of all tested nanoparticles. The absorbance intensity of *A*_400_/*A*_290_ represents the extent of glucose oxidation by Au species and subsequent H_2_O_2_ disproportionation catalyzed by CeNPs. (b) End point assay at 2 min showing the extent of glucose oxidation, calculated as the ratio between cerium peroxo/hydroperoxo complex formation and cerium oxide concentration. (c) Calculated translational diffusion coefficient of tested nanoparticles at different glucose concentrations. (d) Comparison of the average speed of tested nanoparticles at a glucose concentration of 1 mg mL^–1^. (e) Long-range chemotaxis analysis of the nanoparticles toward a high-concentration glucose source. In this setup, an agarose-gel cylinder was placed at one end of a Petri dish to act as a constant glucose source. Nanoparticles at a concentration of 20 mg mL^–1^ were administered at the center-bottom point of the Petri dish and sampled at different time points to plot a heatmap of NPs distribution. (f) Scheme of chemotaxis setup of a Y-shaped microfluidic channel used for chemotactic studies of nanorobots. Positive chemotaxis of nanorobots will result in higher intensity signals toward the upper region. (g) The experimental setup. The glucose solution was injected into the upper inlet while 5 mg mL^–1^ nanoparticles tagged with fluorescent DiI were injected into the lower inlet. The fluorescence intensity of the flow was recorded by an inverted fluorescence microscope. (h) Averaged flow fluorescent image was recorded for 10 s by the fluorescence microscope. (i) Plot of normalized raw fluorescent intensity profile as a function of lateral position along the width of the channel showed whether a shift for nanoparticles toward 10 mg mL^–1^ glucose/PBS mixture as compared to PBS.

Previously, most glucose-powered nanomotors relied on glucose oxidase-catalyzed reactions to generate motion.^35–38^ However, due to the instability of enzyme components in the biological environment, researchers have been exploring alternatives, with notable success in using Au-based species as replacements for glucose oxidase. Kwon et al. reported a complex “egg-in-nest” Au/Pt structure propelled by glucose for targeted intracellular delivery,^16^ while Zheng et al. used Au NPs to catalyze glucose for H_2_O_2_ generation coupled with NO production to generate phoretic force for the propulsion of the micromotors.^39^ Recently, our group explored the glucose propulsion generated by Au NPs based on the possibility of a neutral diffusiophoresis mechanism. In another parallel study, we further downsized the AuNPs to Au single atoms to explore the possibility of efficient glucose oxidation at extremely low Au loadings (as low as ∼1 at %) while still generating sufficient propulsion. Reducing the size of Au species to nanoclusters and single atoms, while simultaneously generating sufficient propulsion via catalyzed glucose oxidation to enhance diffusion, offers a significant advantage by reducing nanorobots’ cytotoxicity and enhancing their mobility in biomedical applications.

Long-range chemotaxis was tested with a previously established method used for nonbiological colloidal particles.^13^ The chemical gradient is generated by placing an agarose gel presoaked with glucose at the far end of a Petri dish, allowing sufficient time for the gradient to be established. The tested nanoparticles were added to the center of the dish, while samples were collected at different time points and different locations for quantification (Figure 3e). Both Au_1_–CeNPs and TPP-Au_11_–CeNPs responded to the glucose gradient by migrating in swarms toward the higher glucose concentration region as far as 0.75 cm in 5 min, while CeNPs diffused gradually with no apparent chemotactic behavior. Due to the extremely small size of these nanoparticles, they would reorient rapidly, making it highly unlikely that chemotactic behavior is generated by phoretic torque to correct any misalignment between the defined catalytic domain and the gradient.^40^ Rather, it is suspected that the chemical gradient influences the rotational diffusion time, resulting in accelerated chemokinesis and rapid dispersion.^41,42^ It needs to be pointed out that using a hydrogel-induced concentration gradient to study chemotaxis has been debated since the density-driven flow needs to be carefully considered,^43^ which is difficult to rule out in experimental settings.

Further chemotactic behavior of gold-based nanomotors was tested by using a Y-shaped microfluidic channel with a fluorescence imaging setup to characterize nanomotor movement along a substrate concentration gradient.^44^ One inlet of the microfluidic channel was injected with nanoparticles tagged with DiI fluorescent probes, while the other inlet was injected with a glucose/PBS solution to generate a chemical gradient (Figure 3f,g). The lateral spreading of the nanoparticles at the region of interest (ROI) at 1 cm down the channel was measured in the presence and absence of glucose. There is a gradient of nanomotors’ fluorescent intensity (Figure 3f), which depends greatly on the location of the ROI. The intensity plots were plotted to calculate the shift at a fixed value of fluorescence intensity as previously reported.^45,46^ A lateral shift was observed for Au_1_–CeNPs and TPP-Au_11_–CeNPs nanomotors toward the region of the channel containing glucose as compared with no substrates, while such a shift was absent in the case of CeNPs (Figure 3h). The shift was further quantified, showing a lateral shift of ∼98 μm for Au_1_–CeNPs and ∼173 μm TPP-Au_11_–CeNPs (Figure 3i).

The above results suggest that the Au-based nanorobots, in the form of either single atoms or nanoclusters, act as nanomotors exhibiting self-propulsion and directional positive chemotaxis in the presence of glucose. This is possibly due to the neutral self-diffusiophoresis generating an asymmetrical slip velocity.^16^ It is also noted that such enhanced active Brownian motion does not necessarily need a Janus structure,^47^ because atomic-level heterogeneity is sufficient to generate such self-propulsion.^48–50^ Single-atom engineering represents a transformative strategy because it maximizes catalytic atom utilization while imparting molecular-scale uniformity and stability, features that are critical for shrinking microrobotic systems into a nanoscale regime. Furthermore, by employing industrially relevant supports such as cerium oxide to immobilize Au single atoms or clusters, this platform provides a realistic pathway for scalable production with a preserved atomic precision and biomedical stability. Owing to their ultrasmall size and active propulsion, these nanorobots traverse the vitreous and reach the retina within ∼ 30 min, whereas larger nanoparticles (e.g., 340 nm) require several hours. This clear size-dependent advantage facilitates their potential for rapid and precise ocular delivery.

### Navigation in Complex Fluids: Controlled Propulsion in Vitreous Humor

Investigating how these ultrasmall nanorobots overcome the barrier of vitreous humor for biomedical applications is essential in ocular disease treatment due to the challenges and controversies surrounding efficient drug delivery to the retina. The vitreous consists of 90% water with solid components like collagen and glycosaminoglycan. The collagen network with a mesh size of about 500 nm is essential for maintaining the vitreous shape. Hyaluronic acid, an anionic polymer, is the predominant glycosaminoglycan present in the vitreous. The combination of these two major components generates the viscoelastic nature of vitreous and provides regulation of particle diffusion through electrostatic interactions.^51^ Glucose serves as a vital component of energy supply in the vitreous,^52^ with an average concentration of 0.5 mg mL^–1^.^53^ Thus, it is inherently motivating to investigate whether glucose-enhanced diffusion of these sub-10 nm nanorobots can be preserved in the viscous media. To mimic the viscous and electrostatic properties of vitreous humor, we used hyaluronic acid (HA) solutions with varying molecular weights. Two types of HA solutions at 200 mg mL^–1^ were tested at 37 °C mimicking human physiological temperature: one with molecular weight ranging from 8 to 15 kDa (named as 8 kDa HA), and another one ranging from 15 to 30 kDa (named as 15 kDa HA). Figure S5 shows the rheological behavior of the HA solution used here to mimic the vitreous. The loss modulus was higher than the storage modulus in both cases, indicating that HA solutions exhibit more viscous than elastic behavior. The 8 kDa HA solution exhibited a viscosity of approximately 40 mPa·s at 37 °C, closely resembling the cytoplasmic viscosity,^54^ while the 15 kDa HA solution has a viscosity of around 400 mPa·s at 37 °C, close to the liquid phase viscosity of vitreous humor.^8,55–57^ Figure 4a,b shows translational Brownian diffusion coefficients of tested particles in both HA solutions with or without glucose, tested at 37 °C. The diffusion parameter was quantified as the translational (*D*_t_) diffusion and rotational (*D*_r_) diffusion coefficients. *D*_t_ and *D*_r_ are related to viscosity via the Stokes–Einstein law, *D*_t_ = κ × *T*/6 × π × η × *r*, and the Stokes–Einstein Debye law, *D*_r_ = κ × *T*/8 × π × η × *r* ^3^, where η is the viscosity of the HA solutions, κ is the Boltzmann constant, and *r* is the particle radius. The CeNPs showed no enhancement of diffusion in the presence of glucose, ranging from 2 to 10 mg mL^–1^. On the contrary, the Au-based nanorobots exhibited observable glucose-dependent diffusion enhancement. Au_1_–CeNPs showed an enhancement of 36% and TPP-Au_11_–CeNPs exhibited an enhancement of up to 26% in the most viscous glucose/HA solutions. Previous studies have shown that functionalizing nanoparticles with anionic polymer coatings can enhance their diffusion, preventing them from being trapped by polyanionic-based glycosaminoglycans. This suggests a potential strategy for improving drug delivery efficiency in the vitreous.^58^

**Figure 4:**
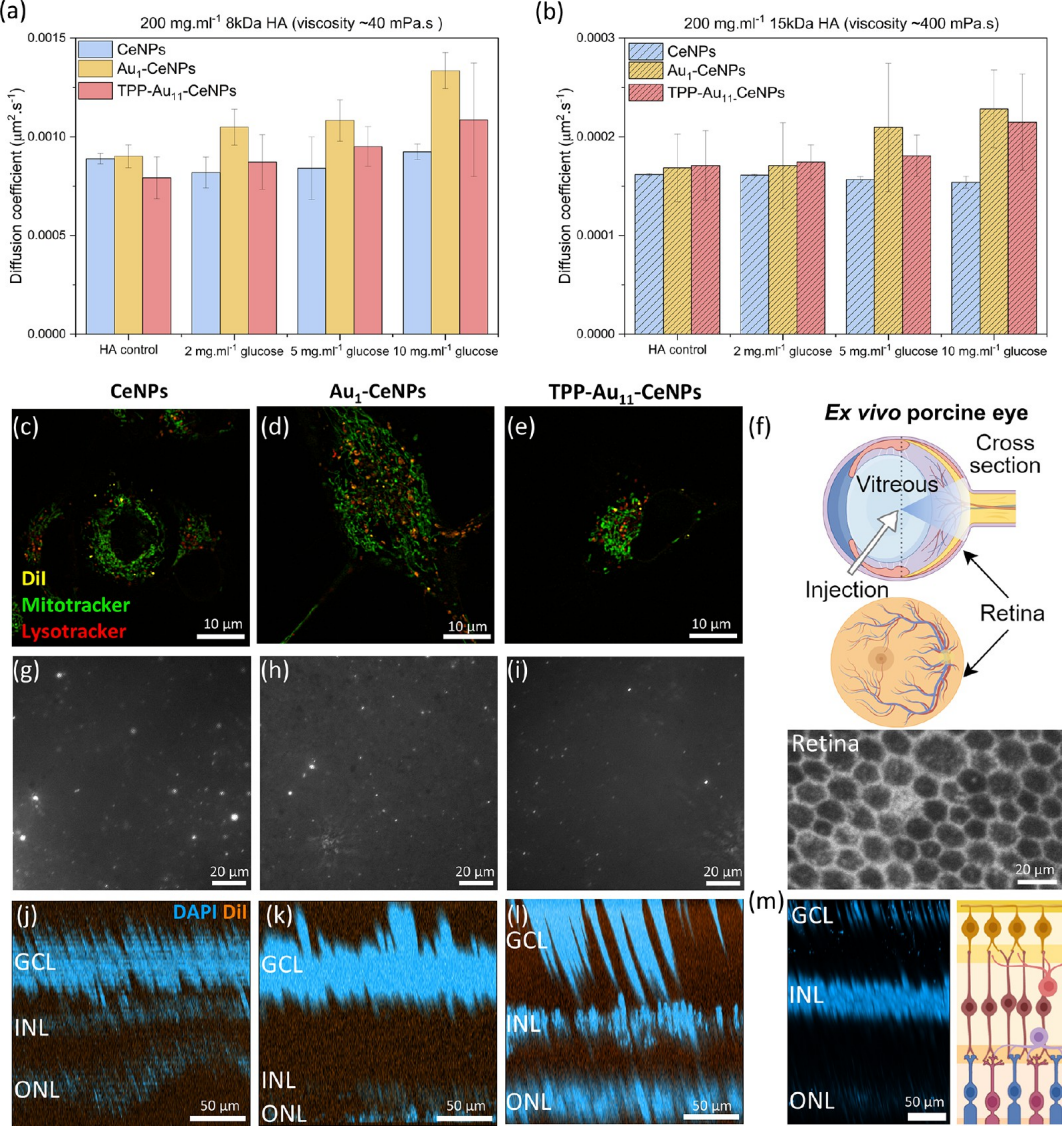
Ex vivo and in vitro tests of nanorobots navigation in complex fluids in the vitreous humor and the cells. (a) Diffusion coefficient of three types of NPs measured in 200 mg mL^–1^ hyaluronic acid (8 kDa) at 37 °C in the presence of glucose ranging from 2 to 10 mg mL^–1^. (b) Diffusion coefficient of three types of NPs measured in 200 mg mL^–1^ hyaluronic acid (15 kDa) at 37 °C in the presence of glucose ranging from 2 to 10 mg mL^–1^. (c–e) Confocal microscopic images of nanorobots internalized into the HT1080 cells after 30 min of coincubation in vitro, with yellow indicating NPs (DiI dye), green indicating mitochondria (Mitotracker Green FM) and red indicating lysosome (Lysotracker Deep Red). (c) DiI tagged CeNPs, (d) DiI tagged Au-CeNPs, and (e) DiI tagged TPP-Au_11_–CeNPs. (f) Schematic illustration of intravitreal injection into ex vivo porcine eyes (horizontal cross-section) and an indication of the retina position measured after dissection. Fluorescent image of retinal cones and rods photoreceptor cells close to fovea sites showing autofluorescence. The scheme is created with BioRender.com. (g) Detected DiI-tagged CeNPs, (h) DiI-tagged Au_1_–CeNPs, and (i) DiI-tagged TPP-Au_11_–CeNPs (bright dots) on the measured retinal layer by epifluorescence microscope measured on the dissected retina after 30 min of intravitreal injection on ex vivo porcine eyes. (j–m) Two-photon excitation microscopy 3D projections of the retinal layers after ex vivo intravitreal injection with three types of nanoparticles, projected along the *XZ* plane to visualize depth distribution. Original 3D distribution and sample preparation are shown in Figure S9. Nuclei were stained with DAPI (blue), and NPs were tagged with DiI (orange). (j) Retina with DiI-tagged CeNPs, (k) retina with DiI-tagged Au_1_–CeNPs, (l) retina with DiI-tagged TPP-Au_11_–CeNPs, and (m) retina without particles as a control. The scheme is created with BioRender.com. The illustration indicates the ganglion cell layer (GCL), composed primarily of ganglion cells; the inner nuclear layer (INL), consisting mainly of bipolar, amacrine, and horizontal cells; and the outer nuclear layer (ONL), which contains the nuclei of photoreceptor cells.

To investigate the motion behavior of nanorobots within a cellular environment, we utilized confocal laser scanning microscopy (CLSM) to gain insights into the dynamics of nanorobots’ interaction with cells in vitro. Fluorescently labeled nanorobots were coincubated with HT1080 cells, a model type of epithelial cells derived from the connective tissue of human fibrosarcoma. After washing off excessive and surface-adsorbed NPs, the cells were stained by Mitotracker and Lysotracker dyes, indicating the location of mitochondria and lysosomes. The NP dosages used in cellular uptake experiments were tested beforehand to ensure they were noncytotoxic (Figure S8). Figure 4c–e confirms the successful cellular uptake of these ultrasmall NPs by the cells, highly probably through the endocytosis pathway as investigated previously by Singh et al.^61^ Incubation of cells with samples for only 30 min already resulted in visible accumulation of NPs inside the cytosol. Observations at higher magnification indicated that the NPs were in the cytoplasm and showed significant colocalization with lysosomes. This finding is consistent with previous studies demonstrating NP localization in the cytoplasm and endosomes/lysosomes, depending on NP size, surface charge, and other factors.^62^ As shown in Supporting Videos S1–S3, the internalized NPs were actively transported within the cells over an observation time scale of a few hours. The mobility of the nanorobots within the cellular environment was challenging to distinguish using confocal or light microscopy, as their movement may be attributed to passive transport via subcellular organelles rather than independent diffusion. Therefore, establishing whether the nanorobots maintain an enhanced diffusion requires further investigation beyond this imaging approach. Supporting Videos S3 shows that some TPP-Au_11_–CeNPs-based nanorobots colocalized with mitochondria, suggesting that TPP may act as a mitochondrial-penetrating ligand, as previously reported.^28^ Although the mitochondrial targeting effect for more efficient ROS scavenging is not conclusively proven by the colocalization data in the case of TPP-Au_11_–CeNPs nanorobots (due to the extremely low amount of TPP), it is promising to further investigate the incorporation of TPP functional ligands onto the surface coatings of nanorobots. This approach could enhance targeting efficacy and is a valuable direction for future research.

To study the propulsion of nanorobots in a more realistic model, we used resected porcine eyes from sacrificed pigs as an ex vivo model. Fluorescently tagged nanorobots were intravitreally injected into porcine eyes (Figure 4f). Following intravitreal injection, the NPs were allowed to diffuse for 30 min before dissecting the eye to obtain the retinal layer of the posterior segment. The fluorescence intensity was normalized for all samples based on the UV–vis spectra of tagged DiI dyes (Figure S6) before injection, and nanorobots were tracked using an inverted epifluorescence microscope. As depicted in Figures 4f and S7, intact photoreceptors such as cones and rod cells can be observed on the retinal segment, while Figure 4g–i show the presence of injected fluorescent tagged NPs reaching the retinal layer. It needs to be pointed out that we observed fluorescent signals of all types of injected NPs that can reach the retinal layer after 30 min of administration, indicating they can diffuse through the vitreous and reach the retina (travel distance ∼ 1 cm). The delivery time is 10 times shorter than that due to the passive diffusion of particles, since it has been reported that 340 nm albumin/HA particles need 6 h to cover ∼1 cm.^59^ To verify the localization of fluorescently tagged nanorobots within the retinal tissue, ex vivo porcine eyes were examined 24 h after intravitreal nanoparticle injection. The retinal layers were isolated, washed, and stained with DAPI to mark nuclear positions, followed by two-photon excitation microscopy 3D imaging. The use of two-photon excitation microscopy provides superior tissue penetration and minimizes photobleaching, enabling high-resolution, three-dimensional visualization of fluorescent signals within thick biological samples such as the retina. As shown in Figure S9, the 3D reconstruction of the retinal section revealed three distinct nuclear layers corresponding to the ganglion cell layer (GCL), inner nuclear layer (INL), and outer nuclear layer (ONL), with the GCL oriented toward the vitreous side. The 3D projections were subsequently analyzed to determine the spatial distribution of nanoparticles within these layers (Figure 4j–m). DiI fluorescence from all three nanoparticle types was detected exclusively within the retinal tissue, spanning the GCL, INL, and ONL, and extending into the photoreceptor region. Notably, TPP-Au_11_–CeNPs exhibited the highest signal intensity near the GCL (Figure S9i,k), suggesting preferential accumulation at the inner retinal surface adjacent to the vitreous, consistent with effective intravitreal delivery and interaction with ganglion cells.

Quantification of the fluorescence intensity to compare the number of NPs reaching the retina is difficult. Despite using freshly resected ocular samples within 4 h of animal sacrifice, vitreous liquefaction under ex vivo conditions is inevitable, leading to a degree of deformation caused by the liquefaction and the presence of aqueous vitreous humor.^60^ The observed results suggest that these ultrasmall nanoparticles can partially overcome the hindrances posed by the vitreous body to reach the retinal layer. The model of artificial vitreous content mimicked by hyaluronic acid and ex vivo porcine eyes both possess advantages and disadvantages. While hyaluronic acid can be used to study the impact of viscosity on nanorobot motion in a more controlled medium, ex vivo porcine eyes with vitreous humor offer a more realistic biological model. However, the post-mortem liquefied aqueous humor of the ex vivo model may affect the motion behavior, presenting some challenges when compared to the in vivo models.

### Intravitreal Application of Glucose-Powered Nanorobots into the Healthy Eye In Vivo

To evaluate the therapeutic effects of glucose-powered nanorobots in reducing retinal degeneration, we first examined their effects on healthy mice. Using an in vivo mouse model, we assessed the efficiency of nanorobot delivery to the retina. The left eye of the mice was intravitreally injected with fluorescently tagged nanoparticles (in a dose of 0.05 or 0.5 μg). As a control, untreated mice or mice with intravitreally injection with PBS were used. The retina, the remaining posterior segment (excluding the retina), and the submandibular lymph nodes were collected on days 1 and 7 after intravitreal application, and the number of NP-positive cells was analyzed by flow cytometry. Figure 5a,d demonstrate that approximately 0.1% of the analyzed retinal cells were infiltrated with nanorobots at higher nanoparticle injection dosages (0.5 μg). Additionally, there was a dosage-dependent relationship concerning the final quantities of nanoparticles reaching the retina. Furthermore, the Au-based nanorobots have higher uptake by retinal cells compared to controlled CeNPs, which could be attributed to the enhanced motion of the endogenous glucose in the tissue (∼0.5 mg mL^–1^).^63^ Figure 5b,e show that up to 15% of posterior segment cells are infiltrated by nanoparticles on day 1, with levels decreasing by day 7. We observed that only a small number of NPs (∼0.02% of detected NP-positive cells) accumulated in the submandibular lymph nodes (Figure 5c,f), which may result from the body’s natural drainage and immune response systems.

**Figure 5:**
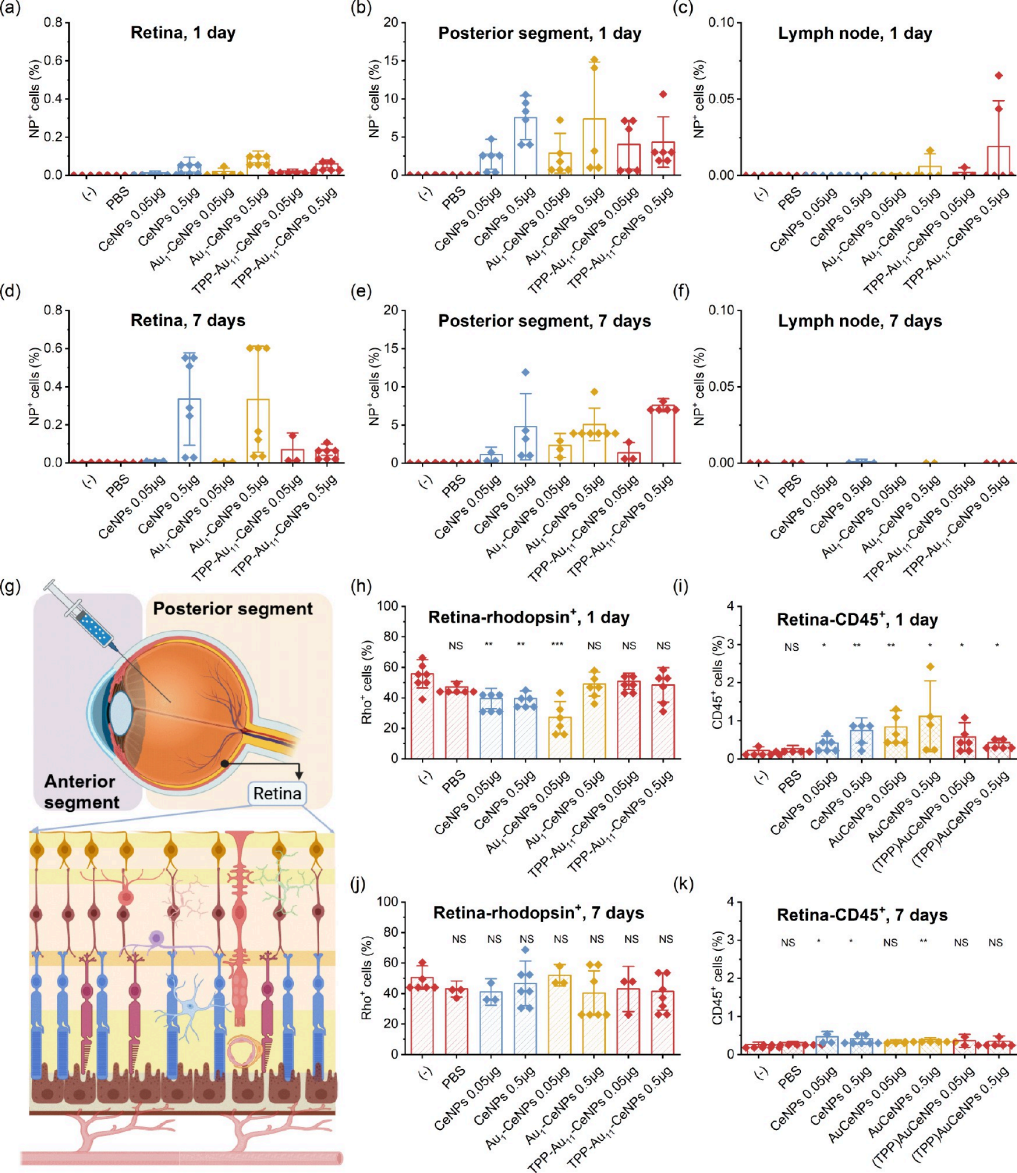
In vivo tests of nanorobots’ effect on the retina in the healthy mouse model. Positive cell percentage with NPs when intravitreally injected with two different dosages and compared with nontreated control and PBS-injected control: (a) retina after 1-day treatment; (b) posterior segment after 1-day treatment; (c) lymph nodes after 1-day treatment; (d) retina after 7-day treatment; (e) posterior segment after 7-day treatment; and (f) lymph nodes after 7-day treatment. (g) Schematic illustration of intravitreal injection into mouse eyes toward the retina (vertical cross-section of the eye and retina). The scheme is created with BioRender.com. (h) Positive rhodopsin cell percentage in the retina after 1 day of NPs intravitreal treatment. (i) CD45^+^ cell percentage in the retina after 1 day of NPs intravitreal treatment. (j) Positive rhodopsin cell percentage in the retina after 7 days of NPs intravitreal treatment. (k) CD45^+^ cell percentage in the retina after 7 days of NPs intravitreal treatment. The number of tested mice in each group is *n* ≥ 6. Data were presented as mean ± s.e.m. *P* values were analyzed by a two-sample *t* test, where NS represents nonsignificant, *P < 0.05, **P < 0.01, ***P < 0.001, and ****P < 0.0001.

Evaluating the effect of NPs on normal retinal function after intravitreal injection is crucial to ensure the treatment does not induce toxicity or impair retinal (e.g., photoreceptor) function (Figure 5g). Figure 5h,j show that there is no statistically significant damage to the photoreceptors (rhodopsin-positive cells) on days 1 to 7 after the intravitreal application with Au_1_–CeNPs and TPP-Au_11_–CeNPs at high dosages, although slight damage is observed after 1 day of CeNPs treatment, which was recovered after 7 days. The extent of immune cell infiltration, indicated by the local accumulation of the CD45^+^ cell (Figure 5j,k), shows that the introduction of foreign NPs caused a minimal immune response on day 1, which returns to normal after 7 days.

Fischer’s group proposed that two major criteria should be fulfilled for the nanomotors to move within the vitreous: (1) particle sizes smaller than the macromolecular network; and (2) reduced interaction between the propellers and the biopolymer network.^8^ The nanorobots designed here have a spherical shape with a diameter of less than 20 nm, which is much smaller than the mesh size of the vitreous (∼500 nm).^64^ The ultrasmall size of these nanorobots ensures their relatively unhindered movement through the biopolymeric network of the vitreous structure. The approach reported herein outperformed delivery methods based on passive diffusion in several aspects. First, active delivery promises lower side effects and higher efficiency with the proposed technology. Second, the experimental results suggest that the particles can move from the center of the vitreous to the retina in less than 30 min. Our in vivo study demonstrated that intravitreal injection of these anionic nanoparticles effectively reaches the retinal cells, with uptake increasing in a dose-dependent manner. Au-based nanorobots showed higher retinal cell uptake compared to nonmotile CeNPs, possibly due to enhanced motion facilitated by endogenous glucose. However, there are no specific target cells for particle uptake. Given the importance of protecting multiple cell types in retinal diseases, such broad infiltration may be beneficial. The interaction between different retinal cells implies that the protection of one cell type can contribute to the survival of others.^3,65^ This broader focus on retinal cells, rather than solely one type, could enhance the potential clinical utility of nanorobot-based therapies. Our in vivo studies indicate that the nanorobots primarily accumulate in the retina with minimal off-target distribution and are partially cleared over time, demonstrating low risk of acute toxicity over the midterm. Importantly, long-term or chronic exposure has not yet been evaluated, representing a compelling avenue for future investigation. We highlight the need for further studies on the long-term biodistribution, degradation, and safety of ocular-targeted nanorobots, which will be critical for their translation to clinical applications.

### Intravitreal Application of Nanoparticles into the Eye with the Degenerated Retina

To assess the possible therapeutic potential of CeNPs, Au_1_–CeNPs, and TPP-Au_11_–CeNPs in the treatment of the degenerated retina, we used an experimental mouse model of retinal degeneration induced by the intraperitoneal application of sodium iodate (NaIO_3_) (in the dose of 40 mg kg^–1^ of body weight). The application of NaIO_3_ provides a useful model of retinal degeneration based on the selective damage of retinal pigmented epithelium (RPE) cells and photoreceptors.^66–69^ NPs were intravitreally injected on day 1 after the induction of degeneration, and the retinas were analyzed on day 7 after the intravitreal injection (Figure 6a). As described before,^69^ degenerated retinas were infiltrated with the immune cells. Figure 6b shows the representative dot plots of CD11b^+^ and CD11b^+^ CX3CR1^+^ double-positive cells (microglia/macrophages) in the retina on day 7 after the application of NaIO_3_. In the retina, CD11b^+^ cells are typically microglia and macrophages involved in immune responses, tissue repair, and inflammation. When these cells also express CX3CR1, they play a role in regulating inflammation and cell migration, which is important for retinal immune surveillance and maintaining homeostasis. This subset of immune cells is critical in responding to retinal injury or disease while balancing tissue integrity.

**Figure 6:**
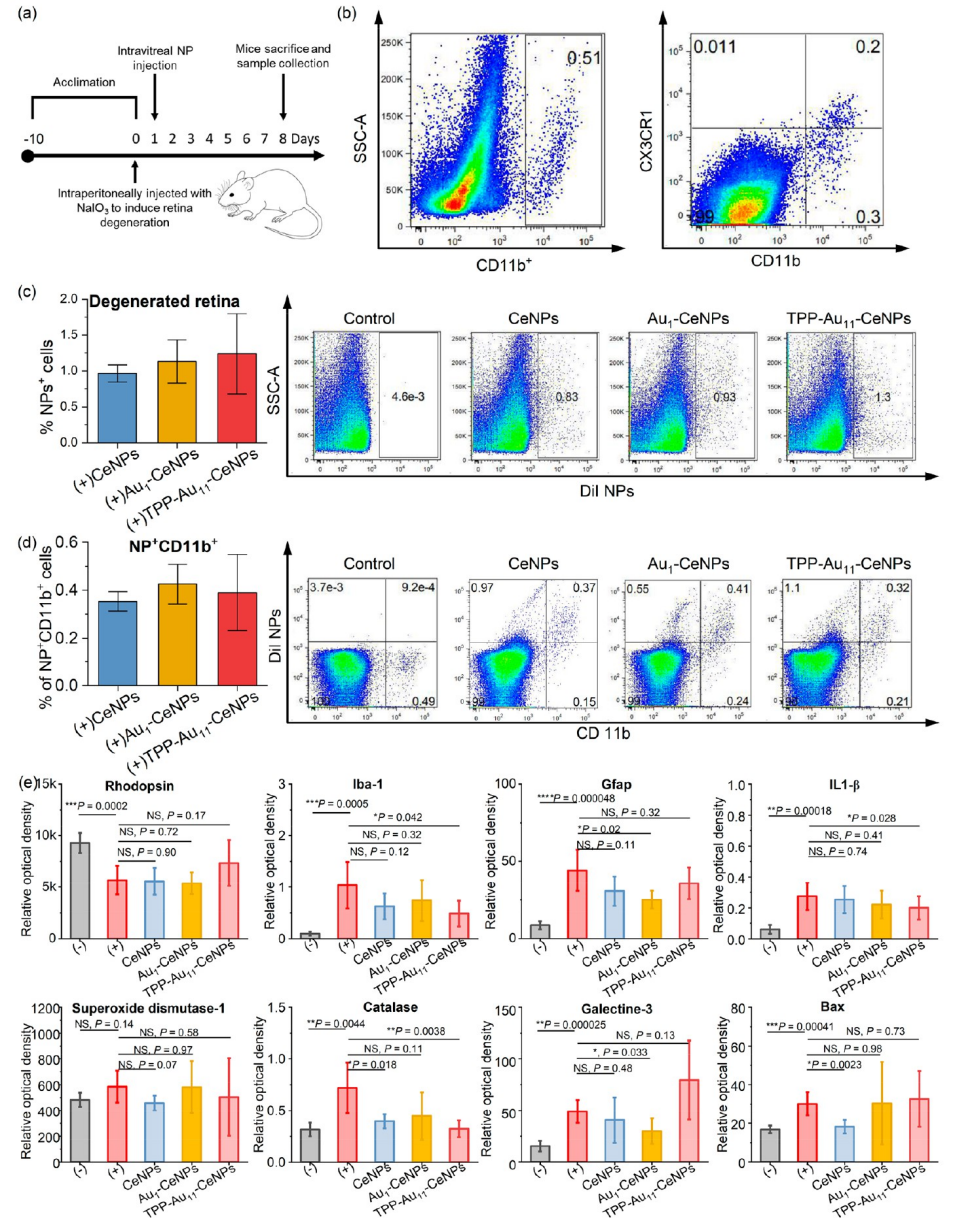
Effect of in vivo application of NPs into the degenerated retina. (a) In vivo acute retinal degeneration was established by intraperitoneal injection of NaIO_3_ into healthy mice after 10 days for acclimation (counted as day 0) to induce selective damage of RPE cells and photoreceptors (rhodopsin-positive cells). On day 1 after intravitreal injection, tested NPs were intravitreally applied into the eye with degenerated retina. On day 8 (7 days after NPs applications), mice were sacrificed, and retinal samples were harvested for further analysis. (b) Representative density dot plots of infiltration of the degenerated retina with CD11b^+^ cells and CD11b^+^ CX3CR1^+^ cells (microglia/macrophages). (c) Infiltrations of degenerated retinas with different types of NPs. (d) Number of NPs^+^ CD11b^+^ double-positive cells in the degenerated retinas. (e) Expression of genes for *rhodopsin*, *Iba-1*, *Gfap, IL-1*β, *superoxide dismutase-1*, *catalase*, *Galectine-3*, and *Bax* were assessed by real-time PCR in the healthy retina (−), degenerated untreated retina (+), and degenerated retina (+) treated with CeNPs, Au_1_–CeNPs, and TPP-Au_11_–CeNPs. The number of mice in each group is *n* ≥ 10. Data were presented as mean ± s.e.m. *P* values were analyzed by a two-sample *t* test, where NS, nonsignificant, *P < 0.05, **P < 0.01, ***P < 0.001, and ****P < 0.0001.

We observed that approximately 1% of the retinal cells were infiltrated with the NPs on day 7 after the intravitreal applications. As in the case of the applications of NPs into the healthy eye, the Au-based nanorobots had 30% higher uptake by retinal cells compared with the controlled CeNPs (Figure 6c). These results further confirm that enhanced diffusion allows nanorobots to accumulate and be taken up by retinal cells more effectively than nanoparticles without motion. In addition, the uptake of NPs by retinal cells was higher in the degenerated retina compared to the healthy retina (1% compared to 0.1%). This effect could be caused by the infiltrated immune cells, mainly microglia and macrophages, with phagocytic activity. It is shown in Figure 6d that 0.4% of CD11b^+^ cells were also positive for NPs, indicating the phagocytosis of nanoparticles by microglia/macrophages. In the retina, glucose transporters facilitate the passive movement of glucose from the blood to retinal cells,^70^ with higher glucose concentrations in the blood compared to the vitreous humor. This gradient enables glucose to diffuse through the retinal pigment epithelium into the photoreceptor layer, where it is absorbed by photoreceptor cells, allowing efficient glucose uptake.^70^ We hypothesize that this glucose concentration gradient plays a key role in guiding glucose-powered nanorobots to accumulate in the retina. The differential glucose distribution across the retinal layers may influence nanorobot movement, directing them toward regions with higher glucose concentrations for targeted therapeutic effects.

The potential effect of intravitreal applications of NPs on the degenerated retina was assessed by the changes in the expression of selected genes (Figure 6e). Specifically, we analyzed genes representing key biological processes associated with retinal degeneration, including photoreceptor integrity (*rhodopsin*), apoptosis (*Bax*), inflammation and microglial activation (*Iba-1*, *Gfap*, *IL-1*β, and *Galectin-3*), and oxidative stress response (*superoxide dismutase-1*, *catalase*). We observed that each of the tested NPs (CeNPs, Au_1_–CeNPs, and TPP-Au_11_–CeNPs) has a different therapeutic effect on degenerate retinal cells. The expression of the gene for rhodopsin (photoreceptor marker) decreased after the application of NaIO_3_, as an effect of retinal degeneration. The intravitreal injection of tested CeNPs and Au_1_–CeNPs had no significant effect on the expression of this gene, but there was a considerable increase in the TPP-Au_11_–CeNPs-treated group, improving retinal degeneration. In general, the therapeutic effects and regulation of genes involved in the protection of retinal cells (*rhodopsin*, *Bax*), immune reaction (*Iba-1*, *Gfap*, *IL-1*β*,* and *galectin-3*), and oxidative stress (*superoxide dismutase-1*, *catalase*) were dependent on the types of NPs. Notably, CeNPs primarily decreased proapoptotic gene *Bax* (*Bax*↓), while Au_1_–CeNPs attenuated neuroinflammation through the downregulation of *Gfap* and *Galectin-3*. TPP-Au_11_–CeNPs demonstrated the strongest multipathway response, characterized by enhanced *rhodopsin* expression (rhodopsin↑), indicating photoreceptor preservation, and suppression of inflammatory (*Iba-1*, *IL-1*β, *TNF-*α) and oxidative stress markers (*catalase*, *SOD1*), reflecting reduced microglial activation and restored redox homeostasis. These gene-level modulations correlate with the observed therapeutic effects, namely, photoreceptor protection, reduced neuroinflammation, and improved retinal microenvironment stability, consistent with the dual antioxidant and immunomodulatory activities of the glucose-powered nanorobots. The latter effect is further investigated in vitro in the following part through the interaction of nanorobots with primary immune cells isolated from bone marrow.

### In Vitro Immunomodulatory Effects of Au-Based Nanorobots

The activation of immune reactions and proinflammatory responses represents one of the leading causes of the development of retinal degeneration.^3,4^ We have shown that a significant proportion of applied NPs was phagocyted by CD11b^+^ cells (Figure 6d). However, Figure 6e shows that injection of NPs regulated the local immune reaction in the retina (regulation of expression of genes for *Iba-1* and *Gfap*). For this reason, we further tested the immunoregulatory potential of NPs on activated CD45^+^ CD11^+^ cells. CD45^+^ CD11^+^ cells are immune cells, typically macrophages or dendritic cells, that play a role in inflammatory responses and are often found in tissues undergoing immune activation or pathology. These CD45^+^ CD11^+^ cells were isolated from mouse bone marrow and separated via antibody-conjugated microbeads (Figure 7a). Representative histograms (Figure S10) show the phenotypic characterization of the CD11b^+^ and CD45^+^ population isolated from the bone marrow. The cells were positive for both CD45 and CD11b, markers characteristic of the microglia/macrophage population. As shown in the graph in Figure 7b, the NPs in the selected doses (125 pg of NPs per cell (pg/cell)) did not affect the viability of the CD11b^+^ CD45^+^ cells. Figure 7c shows representative dot plots of the infiltration potential of NPs into the immune cell population. After 10 min, approximately 3–9% of the cells were already infiltrated with the NPs, and after 24 h, the whole population was already NPs positive. The RT-PCR analysis (Figure 7d) shows that the stimulation of CD11b^+^ CD45^+^ population with proinflammatory cytokines (such as *IFN-*γ, *IL-1*β*,* and *TNF-*α) increased the expression of genes for *IL-1*β, *TNF-*α*,* and *VEGF*. While *IL-1*β promotes inflammation and fever, *TNF-*α mediates cell signaling for inflammation and apoptosis. The *VEGF* gene is crucial for retinal health, but its overexpression can lead to abnormal blood vessel growth and leakage in diseases such as diabetic retinopathy and AMD. *TNF-*α was down-regulated by Au-based nanorobots, and all tested NPs decreased the expression of genes for *IL-1*β and *VEGF.*

**Figure 7:**
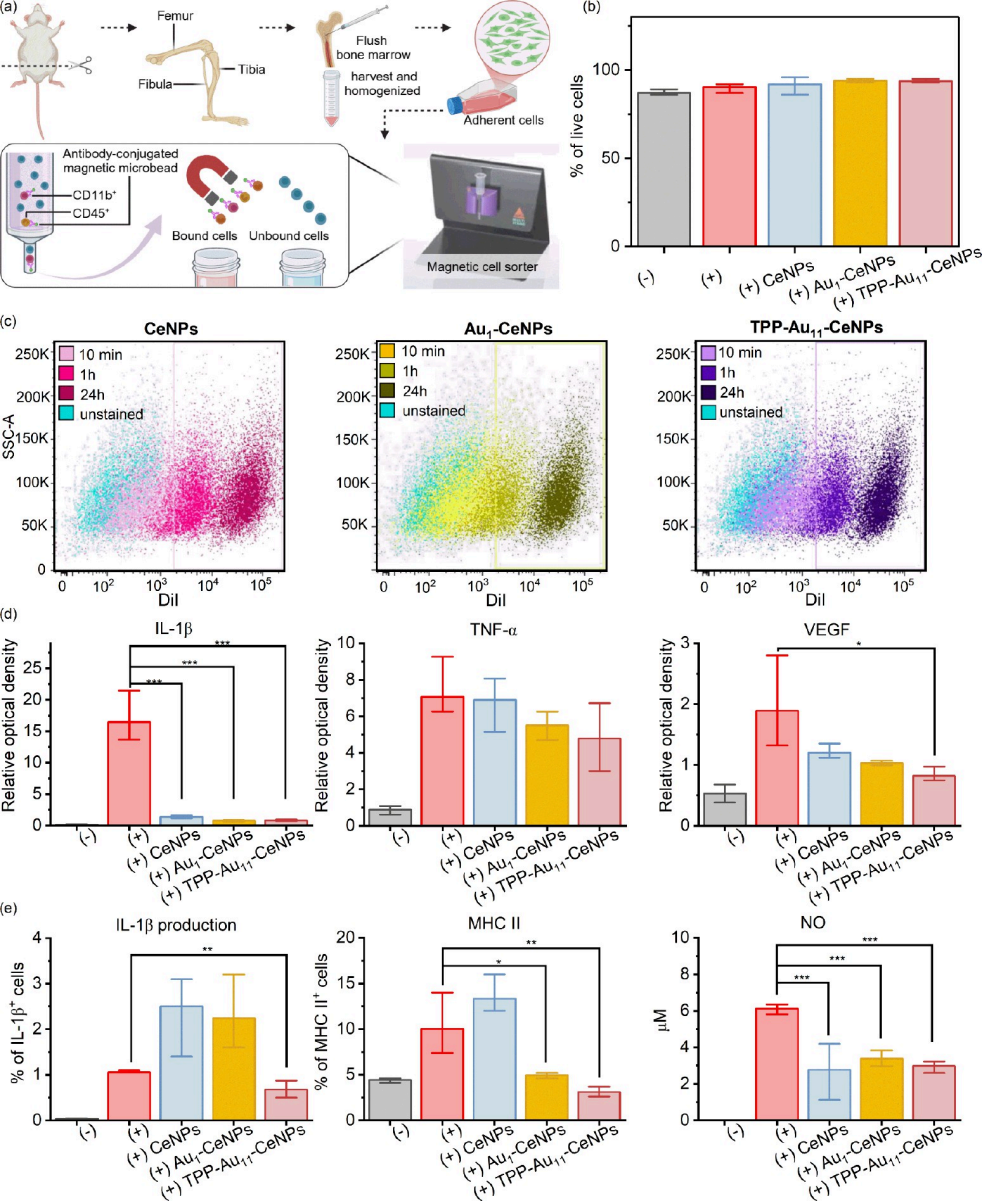
Effect of in vitro nanorobot-immune cell regulation mechanism. (a) Schematic illustration of the purification and isolation of murine-bone marrow derived CD11b^+^ CD45^+^ cells. The scheme is created with BioRender.com. (b) Cell viability of CD11b^+^ CD45^+^ cells incubated with three types of NPs at a concentration of 125 pg NP/cell after 48 h. (c) Representative dot plots of infiltration of the DiI tagged NPs to the CD11b^+^ CD45^+^ cells at different time points at 10 min, 1 h, and 24 h. (d) Expression of genes for *IL-1*β, *TNF-*α, and *VEGF* in the NP-treated CD11^+^ CD45^+^ cells. (e) Protein production of IL-1β and MHC II, and the measured NO production in the NP-treated CD11^+^ CD45^+^ cells. The number in each group is *n* ≥ 3. Data were presented as mean ± s.e.m. *P* values were analyzed by a two-sample *t* test, where NS, nonsignificant, *P < 0.05, **P < 0.01, ***P < 0.001, and ****P < 0.0001.

The regulation of immune cells by Au-based nanorobots was further tested by flow cytometry to analyze the production of proteins (Figure 7e). Production of the IL-1β protein was decreased by TPP-Au_11_–CeNPs, corresponding to the observed downregulation at the gene level to minimize inflammation. However, we do observe an increased level of IL-1β production after cultivation with CeNPs and Au_1_–CeNPs. On the other hand, Au-based nanorobots decreased the surface expression of the MHC II molecule, which is expressed under the stimulation of CD11b^+^ CD45^+^ cells. MHC II molecules are cell surface proteins primarily found on antigen-presenting cells (such as dendritic cells, macrophages, and B cells) that present extracellularly derived antigens to CD4^+^ T cells, initiating immune responses. Under overstimulated inflammatory conditions, suppressed production of MHC II molecules regulated by the Au-based nanorobots can act as a regulatory mechanism to prevent excessive immune activation, potentially reducing retinal tissue damage caused by an overactive immune response. All tested NPs also significantly down-regulated the production of nitric oxide, which has implications that these nanorobots have the potential for modulating inflammatory responses in the examined system.

The retina is an immune-privileged tissue due to the blood-retinal barrier, immunosuppressive factors, and quiescent microglia, which protect the tissue from excessive inflammation.^3^ In retinal degeneration, this privilege breaks down, leading to immune cell infiltration, chronic inflammation, and exacerbated neuronal damage. Overactivation of immune cells in this context can cause uncontrolled inflammation, scarring, and autoimmune responses, worsening retinal degeneration rather than aiding repair. The study demonstrates the strong immunoregulatory potential of Au-based nanorobots in addressing inflammation associated with retinal degeneration. These nanorobots demonstrated targeted interaction with CD11b^+^ CD45^+^ immune cells, effectively reducing the expression of key pro-inflammatory genes such as *IL-1*β, *TNF-*α, and *VEGF* while modulating protein production and surface markers like MHC II to prevent excessive immune activation. Importantly, both in vivo and in vitro analyses confirmed that the Au-based nanorobots are biocompatible, eliciting only transient immune responses while preserving the retinal structure and function. Their ability to downregulate proinflammatory mediators (*IL-1*β, *TNF-*α, and *VEGF*), suppress nitric oxide production, and modulate immune cell activity highlights a unique dual role as both delivery vehicles and immunoregulatory agents for retinal therapy. Furthermore, cerium oxide within the nanorobots contributes synergistically to these effects: in addition to its well-established antioxidant activity as a redox-active catalyst mimicking superoxide dismutase and catalase, CeO_2_ also regulates immune responses by attenuating microglial overactivation and dampening inflammatory signaling. This combined antioxidant and immunomodulatory action was most pronounced in the TPP-Au_11_–CeNPs group, which exhibited the strongest protective and anti-inflammatory outcomes, highlighting the multifaceted role of cerium oxide in driving the therapeutic efficacy of the nanorobots.

## Conclusions

In this study, we demonstrated successful retinal targeting of glucose-fueled, single-atom engineered nanorobots in a murine model of retinal degeneration following intravitreal injection, enabled by their active propulsion and chemotactic behavior through the vitreous humor, and validated using both ex vivo and in vivo imaging techniques. These nanorobots, composed of gold single atoms or nanoclusters within a sub-10 nm size range, are smaller than the mesh size of the vitreous biopolymeric network. Functionalization with a poly­(acrylic acid) minimizes nonspecific interaction with biopolymers such as collagen, facilitating smooth penetration through the vitreous.

Driven by the natural endogenous glucose concentration gradient from retinal blood vessels, the nanorobots exhibit enhanced diffusion and directional chemotaxis, reaching the retina within 1 h. Comparative studies show that both gold single atoms and nanoclusters exhibit similar chemotactic motion, governed by a neutral self-diffusiophoresis mechanism, analogous to glucose oxidase-based micromotors. This active motion promotes efficient cellular uptake and posterior retinal accumulation, supporting their role as precise delivery vehicles.

Fascinatingly, we found that the chemotactic nanorobots themselves exhibit compelling dual therapeutic potential. They can modulate the retinal microenvironment by downregulating pro-inflammatory cytokines (*IL-1*β*, TNF-*α*, VEGF*), suppressing nitric oxide production, and reducing oxidative stress, collectively mitigating retinal degeneration. Their immunomodulatory activity includes regulation of immune cell activation and MHC II expression, contributing to immune homeostasis. These therapeutic effects are attributed to the inherent redox activity of cerium oxide within the nanorobots.

Recent advances in micro/nanorobotics have demonstrated the feasibility of navigating through the vitreous body for targeted ocular drug delivery.^71^ For instance, propeller-shaped nanorobots developed by Wu et al. represent the first example of delivering therapeutic agents to the retina with high precision under magnetic field navigation,^8^ while hybrid biomembrane-functionalized magnetic nanorobots enable penetration of the vitreous for retinal treatments.^19^ It should be noted that both studies relied on magnetically propelled microrobots, which require complex external instrumentation and advanced imaging techniques to track and manipulate these micro/nanoobjects in real time. Such approaches may also pose potential risks to the vitreous structure due to the size of the robots and the strong forces applied. Other studies have explored light-driven microswimmers for controlled motion within the eye.^8,9^ While light is a more biocompatible energy source, none of these studies evaluated potential light-induced damage to retinal cells or photoreceptor function in vivo. In contrast, our study introduces the autonomous propulsion mechanism that does not rely on external fields, combined with a fully biocompatible material platform that enhances both maneuverability and proven safety in the ocular environment.

Moreover, we have recently demonstrated the feasibility of single-atom nanorobots for corneal repair in a mouse model in combination with stem cell therapy.^19^ Unlike our previous work, which relied on stem cells to deliver nanorobots, this study establishes their ability to autonomously traverse the vitreous barrier and directly reach the retinal layer. This represents a significant milestone in advancing sub-10 nm nanorobots as a therapeutic delivery system. At ultrasmall dimensions below 10 nm, particles enter the “quasi-molecular regime,” where dynamic interactions with the endogenous biomolecular environment reduce large-scale cellular and immune recognition events. As a result, these ultrasmall nanorobots can penetrate cellular and biological barriers within tissue architectures in a manner comparable to small-molecule drugs.^72^

Importantly, this work demonstrates that single-atom-engineered nanorobots can independently overcome vitreous barriers and deliver therapeutic functions at the retinal level, highlighting their superior stability, catalytic efficiency, and nanorobotic propulsion compared with previously reported systems. Owing to their ultrasmall size and active propulsion, these nanorobots traverse the vitreous and reach the retina within ∼30 min, whereas larger nanoparticles (e.g., 340 nm) require several hours. Quantitative in vivo analysis demonstrated that ∼0.1% of retinal cells were infiltrated with nanorobots in healthy mice, increasing to ∼1% in retinal degeneration models, where Au-based nanorobots showed ∼30% higher uptake than controls. This clear size-dependent advantage facilitates their potential for rapid and precise ocular delivery. This unique combination of features enables precise and minimally invasive ocular drug delivery, representing a clear advancement over existing microrobotic and nanorobotic strategies. Collectively, our findings provide strong proof-of-concept for chemical-based, chemotactic nanorobots as next-generation platforms, both as intelligent nanocarriers for targeted ocular drug delivery and as standalone therapeutic agents for retinal and other ocular diseases.

## Experimental Section

### Chemicals

Poly­(acrylic acid), cerium nitrate hexahydrate (CeNO_3_)_3_·6H_2_O, 99.99%), gold­(III) chloride trihydrate (HAuCl_4_·3H_2_O, 99.9%), chloro­(triphenylphosphine)­gold­(I) ([(C_6_H_5_)_3_P]­AuCl, 99.9%), tetrahydrofuran (THF, C_4_H_8_O, 99.9%), pentane (CH_3_(CH_2_)_3_CH_3_, 98%), hexane (CH_3_(CH_2_)_4_CH_3_, 95%), dichloromethane (DCM, CH_2_Cl_2_, 99.9%), ammonium hydroxide (NH_4_OH, >25% NH_3_), ethanol (CH_3_CH_2_OH, absolute), methanol (CH_3_OH, 99.9%), Dimethyl sulfoxide ((CH_3_)_2_SO DMSO, 99.9%) sodium borohydride (NaBH_4_, 99%), α-d-glucose (anhydrous 96%), phosphate buffer saline (10× concentrate), hydrogen peroxide (H_2_O_2_, 30% w/w in H_2_O), and agarose were all purchased from Sigma-Aldrich (Merck, Germany). Dil stain (1,1′-dioctadecyl-3,3,3′,3′-tetramethylindocarbocyanine perchlorate), Mitotracker Green FM, and Lysotracker Deep Red were purchased from Thermofisher Scientific. Sodium hyaluronate (hyaluronic acid) was purchased from Contipro a.s. (Czech Republic).

### Synthesis and Characterization of Au-CeNPs Nanorobots

Au_11_(PPh_3_)_7_Cl_3_ was synthesized following a modified protocol based on McKenzie et al.^24^ Au­(PPh_3_)Cl (0.8 mmol) in 20 mL THF was mixed with NaBH_4_ (4 mmol) in 20 mL ethanol. After 2 h of stirring at room temperature, the mixture was precipitated in 400 mL of pentane for another 2 h. The clusters were filtered and washed 4 times with hexane (6 mL each) and 1 time with a 50:50 mixture of DCM/hexane (10 mL each). The precipitate was rinsed with THF until it was colorless. The red precipitate was dissolved in DCM, and the solvent was removed under reduced pressure. For the synthesis of CeNPs and Au-based nanorobots, Ce­(NO_3_)_3_·6H_2_O (2.5 mmol) and poly­(acrylic acid) (0.5 g) were mixed in 13 mL of water. NH_4_OH (15 mL) was also added dropwise into the mixture under continuous stirring. For Au_1_–CeNPs, gold chloride trihydrate (0.25 mmol) dissolved in water (1 mL) and NaBH_4_ (0.125 mmol) dissolved in ethanol (1 mL) were added dropwise into the cerium salt-polymer mixture. For TPP_11_–Au-CeNPs, Au_11_(PPh_3_)_7_Cl_3_ (0.25 mmol) dissolved in methanol (2 mL) was added dropwise into the cerium salt-polymer mixture. The reaction mixture was kept stirring at room temperature for 96 h. Colloidal products were collected by 10 min centrifuge at 5580 × *g* (Sorvall LEGEND X1, Thermo Scientific) after removing the sediments. The supernatant was further centrifuged by an ultracentrifuge (Optima MAX-XP, Beckman Coulter) at 230,000 × *g* for 12 h. Precipitates were collected each time and washed with DI water, repeating ultracentrifugation 3 times. Final precipitates were collected and dispersed in DI water. To perform DiI fluorescent labeling into NPs, 5 mL of 10 mg mL^–1^ nanoparticles (in water) were mixed with 50 μL of 10 mg mL^–1^ DiI (dissolved in DMSO) and stirred continuously for 4 h. Subsequently, any unbound dye was removed by dialysis against deionized water using a 10K MWCO snakeskin dialysis tube (Thermo Scientific) over 24 h.

### Physicochemical Characterization and Motion Characterization

The ATR-FTIR (Vertex 70v, Bruker, Germany) was used to characterize the TPP-Au_11_ clusters. The crystalline structure was determined by XRD using a Rigaku SmartLab 3 kW diffractometer equipped with a Cu Kα anode X-ray tube operated at 40 kV and 30 mA. Surface chemical composition was studied by XPS using a Kratos Analytical Axis Supra instrument with a monochromatic Al Kα (1486.7 eV) excitation source. The survey spectra were collected with a pass energy of 80 eV, a step size of 1 eV, a dwell time of 100 ms, and an emission current of 5 mA. The core-level spectra were collected with a pass energy of 20 eV, a step size of 0.1 eV, a dwell time of 300 ms, and an emission current of 10 mA. All spectra were calibrated to the adventitious C 1*s* peak at 284.8 eV and fitted using KolXPD (kolibrik.net). Light adsorption was measured using a Jasco V-750 UV–visible spectrophotometer in the liquid phase with a quartz cuvette of path length of 1 cm.

The TEM samples were prepared from dispersed NPs in aqueous solutions drop-casted on an ultrathin carbon membrane coated on lacy carbon-covered copper TEM grids (Agar Scientific, UK). The HAADF-STEM was conducted with an image-corrected TITAN Themis 60–300 (Thermo Fisher Scientific, USA) operated at an acceleration voltage of 300 kV and with a beam current of ∼50 pA and under the Z-contrast imaging condition of the used HAADF detector. The presented STEM images were acquired and processed with Velox v2.14 software.

The coupled cascade glucose oxidase and catalase mimicking activities of NPs were monitored by measuring the UV–vis absorbance spectra at 290 and 400 nm, following a similar protocol described previously.^73^ DLS-related hydrodynamic diameter and zeta potential measurements were performed in water and PBS solutions (pH 7.4) using a Malvern Panalytical Zetasizer Ultra instrument. Colloidal solution of NPs in the concentration of 0.025 mg mL^–1^ in PBS solutions (1 mL) was used in addition to different concentrations of glucose, ranging from 1 to 20 mg mL^–1^. The backscattering data with a scattering angle of 173° were collected as intensity-based scattering and transformed into diffusion coefficient and relaxation time based on the Malvern software ZS explorer (V1.3.2.27). For a long-range chemotaxis setup, a Petri dish with a 3.5 cm diameter was filled with 5 mL of PBS (pH 7.4) solution. A 1% agarose gel cylinder 0.6 mm in diameter and 0.5 mm in length was presoaked in 1 M glucose solution overnight. The agarose gel cylinder was placed at the edge of the Petri dish. After placing the cylinder, the Petri dish was kept still without perturbation for 60 min to allow sufficient glucose diffusion. NPs at 20 mg mL^–1^ were introduced at the central point of the Petri dish by a needle syringe. Ten microliters of the samples were withdrawn at different locations at 0 and 5 min and further diluted to a 2 mL volume. The collected samples were analyzed by a UV–vis absorbance spectrometer and subsequently converted to concentration based on CeNPs absorbance at 290 nm.

For the Y-shaped microfluidic channel chemotaxis experiments, Ibidi μ-Slide III 3in1 (uncoated) was used with the middle inlet blocked to generate a two-inlet system. The channels had a total volume of 60 μL and a height of 0.4 mm inside the channels. The flow rate of 1.5 mL h^–1^ was used at each channel to generate a sufficient gradient. The flow rate was controlled by two external Infuse/Withdraw Pump 11 Pico Plus Elite pumps (Harvard Apparatus). At one inlet, 10 mg mL^–1^ glucose/PBS solution (PBS solution only in the case of control experiments) was injected, while at the other inlet, 5 mg mL^–1^ fluorescently tagged NPs were injected. The fluorescence intensity profiles of the flow were recorded by an inverted fluorescence microscope (Nikon ECLIPSE Ti2 Series) equipped with a digital camera (Hamamatsu, C 13440). A TRITC filter cube with an excitation filter of 542/20 nm and an emission filter of 620/52 nm was used with a white-light LED source (CoolLED, pE-300 lite) set at 60% light intensity. Videos were recorded at 25 FPS at 4× magnification, and the intensity profile of flows of 10 s were normalized and analyzed by ImageJ software at set portions of the slides.

Rheological properties of hyaluronic acid solutions were assessed with an advanced rotational rheometer DHR-2 (TA Instruments, USA) at 37 °C isothermal conditions using a 40 mm cone plate geometry. Oscillatory frequency and strain amplitude tests were performed. Frequency sweep tests with a strain amplitude of 1% were conducted across a frequency range of 0.1 to 1 Hz, while amplitude sweep tests were conducted with strain percentages ranging from 0.1 to 1% at a set frequency of 1 Hz. The complex viscosity, storage modulus, loss modulus, and complex modulus were plotted based on both frequency and strain oscillatory tests. The motion of nanorobots in HA solutions was measured by DLS as described above, except that the setting of the solvent viscosity in the software settings was adjusted accordingly.

### Cell Line Preparation and Confocal Microscopy

The cell line HT1080, derived from the connective tissue of human fibrosarcoma, was used in this study. This cell line was obtained from CLS Cell Lines Services GmbH, Germany. The cells were grown in a Gibco minimum essential medium (MEM) supplemented with l-glutamine (2mM), sodium pyruvate (1 mM), and nonessential amino acids (0.1 mM) and incubated in a humidified atmosphere (5% CO_2_ and 95% air) at 37 °C.

The cytotoxicity of nanoparticles was determined using a CellTiter 96 MTS (3-(4,5-dimethylthiazol-2-yl)-5-(3-carboxymethoxyphenyl)-2-(4-sulfophenyl)-2*H*-tetrazolium, inner salt; MTS) assay kit from Promega. HT1080 cell line cell culturing was performed in microplates (5000 cells per well). After the culture medium was removed, solutions of tested nanoparticles were added with increasing concentrations (1–100 μg mL^–1^). For the MTS assay, the solution containing the nanoparticles was removed and replaced with a fresh culture medium containing MTS and incubated for 24 h, and then the absorbance at 490 nm was measured with a 96-well plate reader (SPECTROstar Nano, BMG Labtech, Germany).

For NP cellular uptake experiments, 200,000 cells were seeded in 2 mL of media in Ibidi 35 mm high glass-bottom μ-dishes with replicates for 2 days before NP addition. For NP coincubation, cells in each dish were replaced with media containing 20 μg mL^–1^ of DiI-tagged NPs. After coincubation of cells and NPs for 30 min, the cells were washed with PBS buffer solutions two times and stained with media containing 50 nM Mitotracker Green FM and Lysotracker Deep Red for 20 min. The cells were washed 2 times with PBS buffer and replaced with filtered media (0.22 μm sterile filter) for further confocal microscopy analysis.

The confocal laser scanning microscope (Carl Zeiss Inc., Oberkochen, Germany) was used to acquire confocal microscopy images. For colocalization experiments, Lysotracker Deep Red was excited with the 639 nm solid-state laser (0.1%), while emitted light was detected at 670–723 nm, and MitoTracker Green was excited with a 488 nm argon laser (0.3%) and emitted light was detected at 488–630 nm. DiI-labeled NPs were excited with a 543 nm solid-state laser (0.2%), and emitted light was detected at 540–575 nm. Confocal microscopy-acquired images were analyzed using ImageJ software. Deconvolution was performed with the ImageJ^74^ plugin Iterative Deconvolve 3D, the theoretical PSF for which was generated with Diffraction PSF 3D.^75^

### Ex Vivo Porcine Vitreous Preparation and Propulsion of Nanorobots Reaching the Retina

Porcine eye samples were generously donated by the Veterinary Research Institute of the Czech Republic and the International Clinical Research Center of St. Anne’s University Hospital, Brno, Czech Republic. The ocular samples were dissected from piglets and stored on ice for transportation within 2 h of the piglets’ sacrifice. 100 μL of DiI fluorescent-labeled NPs were intravitreally injected with a 31G needle into each dissected eye and kept on ice for 30 min. After 30 min, the ocular samples were carefully dissected, the vitreous part was removed, and the posterior eyecups were placed inverted onto a glass slide with retinal layers facing down. The samples were analyzed by a Nikon inverted epifluorescence microscope (ECLIPSE Ti2) with a 40× objective. The same TRITC filter cube with an excitation filter of 542/20 nm and an emission filter of 620/52 nm was used with a white-light LED source (CoolLED, pE-300 lite) to detect the fluorescently tagged DiI NPs.

For two-photon excitation microscopy observation of retinal layers, ocular samples were intravitreally injected with 100 μL of DiI-labeled fluorescent nanoparticles and stored at 4 °C for 24 h. After incubation, the eyes were carefully dissected to isolate the retinal layers, which were then fixed in 4% paraformaldehyde (PFA) in PBS for 1 h at room temperature. The fixed samples were subsequently washed three times with PBS (10 min each) to remove residual fixative. Finally, the retinal tissues were stained with DAPI (4′,6-diamidino-2-phenylindole) to visualize nuclei and imaged using a Zeiss LSM 980 confocal laser scanning microscope (Carl Zeiss Inc., Oberkochen, Germany) equipped with a Chameleon Ti: Sapphire Laser 700–1000 nm for two-photon excitation. A 10× Plan-Apochromat objective with NA at 0.45 was employed for image acquisition. For DiI detection, excitation was set at 543 nm with an emission collection at 551 to 569 nm, while for DAPI detection, excitation was set at 720 nm with emission at 353 to 465 nm. Postacquisition, images were deconvolved using a fast iterative Richardson–Lucy algorithm with Poisson statistics and 50 maximum iterations (ZEN Blue software, Carl Zeiss).

### Animals

Female mice of the inbred strain BALB/c (8–15 weeks old), from the company Envigo (Indianapolis, IN, USA), were used in this study. The use of animals was approved by the Local Ethical Committee of the Institute of Experimental Medicine of the Czech Academy of Science, Prague (approval code 7448/2023).

### Intravitreal Application of NPs and Induction of Retinal Degeneration

Mice were anesthetized using an intraperitoneal injection of 0.15 mL of xylazine (xylazinum hydrochloridum 2%, Bioveta, Ivanovice, Czech Republic) and 0.15 mL of ketamine (ketaminum hydrochloridum 5%, Bioveta). The intravitreal application of 0.01 μL of NPs or PBS was performed using a Hamilton syringe (5 μL volume, Hamilton, Reno, NV, USA) with a 33G sharp needle (Hamilton). After the selected period (24 h or 7 days), animals were euthanized, the eyeballs were enucleated; the cornea, the lens, and the vitreous were removed, and the retina was gently dissected from the eyeball. For further analysis, the retina, the remaining posterior segment, and the submandibular lymph nodes were used. For the induction of retinal degeneration, mice were administered a single intraperitoneal injection of NaIO_3_ (Sigma-Aldrich) dissolved in phosphate-buffered saline (PBS) in a dose of 40 mg kg^–1^ of body weight.

### Preparation of the CD11b^+^ CD45^+^ Population

Bone marrow from the femurs and tibias of BALB/c mice was flushed out and homogenized using a tissue homogenizer. Single-cell suspension was seeded in a 75 cm^2^ tissue culture flask (TPP) in RPMI 1640 medium (Sigma-Aldrich) containing 10% FBS, antibiotics (100 U/ml of penicillin, 100 μg/mL of streptomycin), and 10 mM HEPES buffer (referred to as complete DMEM). After 48 h, nonadherent cells were washed out, and the remaining adherent cells were cultivated at 37 °C in an atmosphere of 5% CO_2_ for an additional 2 weeks. The cells were harvested using 1 mL of 0.5% trypsin solution (Sigma-Aldrich), and incubated with CD11b and CD45 MicroBeads (Miltenyi Biotec, Bergisch Gladbach, Germany) for 15 min. The CD11b^+^ CD45^+^ population was separated using a MidiMACS separator with attached LS Columns (both Miltenyi Biotec).

### Cultivation of the CD11b^+^ CD45^+^ Cells in the Presence of NPs

CD11b^+^ CD45^+^ cells were seeded into a 48-well plate (200,000 cells/well) in 1 mL of complete RPMI 1640 medium with TNF-α, IL-1β, and IFN-γ (10 ng/mL) and NPs in a dose of 125 pg of NPs per cell (pg/cell). After a 48 h cultivation, the supernatants were harvested and used for analysis of the production of NO. Cells were transferred into 500 μL of TRI reagent and stored at −80 °C or used for flow cytometry analysis.

### NO Detection and Enzyme-Linked Immunosorbent Assay (ELISA)

The concentration of NO was determined by the Griess reaction in 100 μL of the tested supernatant harvested from the cultivation of CD11b^+^ CD45^+^ cells by adding 100 μL of a mixture of 1% sulphanilamide and 0.3% *N*-1-naphthyl ethylene diamine dihydrochloride (both in 3% H_3_PO_4_). The reaction was quantified using a Sunrise Remote ELISA Reader.

### Detection of Gene Expression by RT-PCR

The total RNA was isolated from the samples by TRI reagent (according to the manufacturer’s instructions). Reverse transcription was performed with deoxyribonuclease I (Promega, Madison, WI, USA) in DNase I buffer (Promega), and the first cDNA strand was synthesized with random primers (Promega) using M-MLV reverse transcriptase (Promega). The total volume of the reaction mixture was 25 μL. Quantitative RT-PCR was performed using SYBR green System (Applied Biosystems, Foster City, CA, USA) by StepOne-Plus Real-Time PCR (Applied Biosystems) with the following parameters: denaturation at 95 °C for 3 min, 40 cycles at 95 °C for 20 s, annealing at 60 °C for 30 s, and elongation at 72 °C for 30 s. Data on fluorescence were collected at each cycle after the elongation at 80 °C for 5 s and analyzed by StepOne Software version 2.3 (Applied Biosystems). Glyceraldehyde 3-phosphate dehydrogenase (GAPDH) was used as a reference gene for the calculation of the relative expression of the analyzed gene. The primers used for amplification are shown in Table S1.

### Flow Cytometry

The retina and posterior segment were digested for 45 min at 37 °C with 1 mg/mL of collagenase I (Sigma-Aldrich) in Hank’s balanced salt solution (HBSS). The submandibular lymph nodes were digested for 1.5 h at 37 °C with 1 mg/mL collagenase II (Sigma-Aldrich) in HBSS.

Single-cell suspension of the retinal cells, posterior segment cells, lymph node cells, or CD11b^+^ CD45^+^ cells were incubated for 30 min at 4 °C with antimouse monoclonal antibodies conjugated with allophycocyanin (APC), phycoerythrin (PE), or fluorescein isothiocyanate (FITC). For the intracellular staining of IL-1β, the cells were cultivated before labeling with Brefeldin A (eBioscience) in a concentration of 10 μg/mL for 2 h. Cells were labeled with live/dead fixable violet dead cell stain kit fixed for 30 min at 4 °C with 100 μL IC fixative buffer and then labeled for an additional 30 min with anti-IL-1β. Antibodies used in experiments are shown in Table S2. Data were collected using an LSRII flow cytometer (BD, Biosciences, Franklin Lakes, NJ, USA) and analyzed by FlowJo 9 or FlowJo 10 (Tree Star, Ashland, OR, USA).

## Supplementary Material









## Data Availability

The authors declare that all data supporting the findings of this study are available within the Article and its Supporting Information. Any additional requests for information can be directed to the corresponding author.

## Notes

Experimental procedures and synthesis of nanoclusters; characterization by UV–vis, fluorescence spectroscopy, and HAADF-STEM; rheological measurements of hyaluronic acid; retina fluorescence imaging; cytotoxicity assays; and flow cytometry details (DOCX)
Time-lapse video showing internalization and intracellular movement of CeNPs in cells (AVI)
Time-lapse video showing internalization and intracellular movement of Au1-CeNPs in cells (AVI)
Time-lapse video showing internalization and intracellular movement of TPP-Au11-CeNPs in cells (AVI)
