# Supplementary material for: Glucose-Powered Ultrasmall Chemotactic Nanorobots for Retinal Degeneration Treatment
Source: J Am Chem Soc. 2025 Nov 21;147(49):45405–22. doi: 10.1021/jacs.5c15651 (PMC12714100; doi:10.1021/jacs.5c15651)
Supplement: Supplementary file 1 [file ja5c15651_si_001.docx]

Supporting Information for

Glucose-Powered Ultrasmall Chemotactic Nanorobots for Retinal Degeneration Treatment

Xiaohui Ju^1^, Kateřina Palacká^2,3^, Roshan Velluvakandy^1^, Jan Michalička^4^, Martin Pumera* ^1,5,6,7^

^1^Future Energy and Innovation Laboratory, Central European Institute of Technology, Brno University of Technology, Purkyňova 123, Brno, 61200, Czech Republic

^2^Department of Toxicology and Molecular Epidemiology, Institute of Experimental Medicine of the Czech Academy of Sciences, Vídeňská 1083, Prague, 14220, Czech Republic

^3^Faculty of Science, Charles University, Albertov 2038, Prague, 12800, Czech Republic

^4^Central European Institute of Technology, Brno University of Technology, Purkyňova 123, Brno, 61200, Czech Republic

^5^Advanced Nanorobots & Multiscale Robotics Laboratory, Faculty of Electrical Engineering and Computer Science, VSB - Technical University of Ostrava, 17. listopadu 2172/15, 70800, Ostrava, Czech Republic

^6^Department of Medical Research, China Medical University Hospital, China Medical University, No. 91 Hsueh-Shih Road, Taichung, 40402, Taiwan

^7^Department of Chemical and Biomolecular Engineering, Yonsei University, 50 Yonsei-ro, Seodaemun-gu, Seoul 03722, Korea

1. **Synthesis and characterization of TPP-Au_11_ nanoclusters**

The synthesis of TPP-Au_11_ nanoclusters (Au_11_(PPh_3_)_7_Cl_3_) was carried out following the previously described methods.^1, 2^ 400 mg of Au(PPh_3_)Cl were dissolved in 20 ml tetrahydrofuran (THF), where 20 ml of 7.6 mg.ml^-1^ sodium borohydride dissolved in ethanol was added dropwise. The reaction mixture was continuously stirred at room temperature for 2 hours. 400 ml pentane was added to the mixture to precipitate the crude product. The precipitates were filtered and washed at least 5 times with a total amount of 30 ml of hexane. A dichloromethane/hexene (10 ml each) mixture was used to wash the precipitates once directly on the filter. The precipitates were further rinsed with around ~500 ml THF until the filtrates came out colorless. The red-colored cluster products were carefully removed from the filter paper and redissolved into 5 ml dichloromethane (DCM), followed by removing the solvent in a vacuum oven with a reduced pressure at RT. The collected products were used for analysis immediately. Otherwise, it was stored under -20 °C to maintain its stability.

The purified TPP-Au_11_ nanoclusters were characterized by UV-visible spectroscopy (UV-vis, Jasco V-750), attenuated total reflection Fourier-transform infrared spectroscopy (ATR-FTIR, Bruker Vertex V70), and X-ray photoelectron spectroscopy (Kratos Analytical Axis Supra) (**Figure S1**). The shape and peak positions at 307, 380, and 420 nm of the UV-vis spectrum in Au_11_(PPh_3_)_8_Cl_2_]Cl are in perfect agreement with those reported by McKenzie^1^ and Truttmann et al., ^2^ ruling out the possibility of side products such as Au_11_(PPh_3_)_8_Cl_2_]Cl since it should shift the UV-vis peaks. ATR-FTIR (**Figure S1b**) also confirmed the presence of peaks at 1478, 1435, 1096, 745, 705, and 690 cm^-1^. The peaks around 3000 cm^-1^ were attributed to residual organic solvents. Figure S1c shows the core level spectra of Au 4*f*, C 1*s*, P 2*p*, and Cl 1*s* corresponding to the elemental presence in the TPP-Au_11_ clusters. It is noted that Au 4*f* of nanoclusters is shifted to a higher binding energy with respect to the bulk Au 4*f* line, which is commonly observed in photoelectron peaks of metal clusters due to an electrostatic final state effect. ^3^ The presence of C 1*s*, P 2*p,* and Cl 1*s* and the absence of O 1*s* also confirms the possibility of this TPP-based structure.


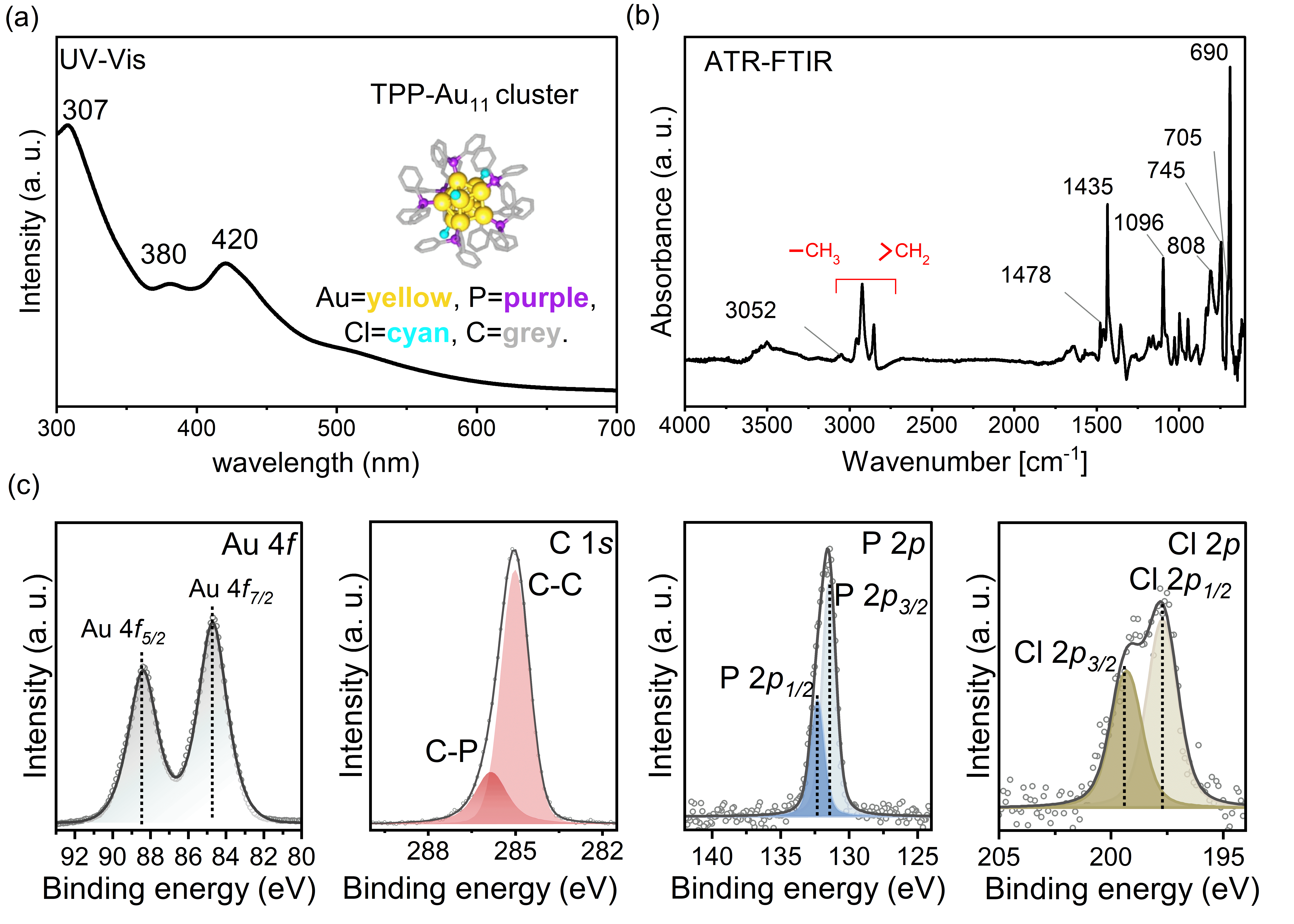


**Figure S1. Characterization of synthesized gold nanoclusters_._ (a)** UV-vis spectra of synthesized TPP-Au11 nanoclusters Au_11_(PPh_3_)_7_Cl_3_ measured in DCM. Structural representation of the cluster is inserted with color code: Au-yellow, P-purple, Cl-cyan, and C-grey, adapted under CC-BY 4.0 license from reference [2], 2022, Wiley. **(b)** ATR-FTIR spectrum. **(c)** XPS core level spectra of Au 4*f*, C 1*s*, P 2*p,* and Cl 2*p* measured by drop-cast of the cluster dissolved in DCM onto a silicon wafer.

**
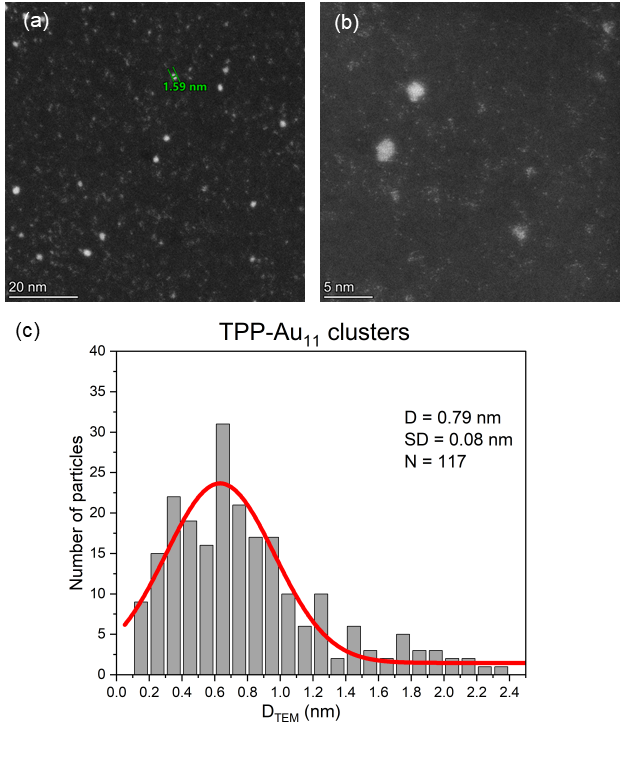
Figure S2. Morphological and size characterization of synthesized gold nanoclusters by HAADF-STEM (High-angle annular dark-field scanning transmission microscope). (a)** and **(b)** are HAADF images. In Figure **(a)** an example of a larger cluster agglomerate is indicated by a measured size of 1.59 nm. **(c)** Particle size distribution and fitted curve of TPP-Au_11_ clusters obtained from STEM images.


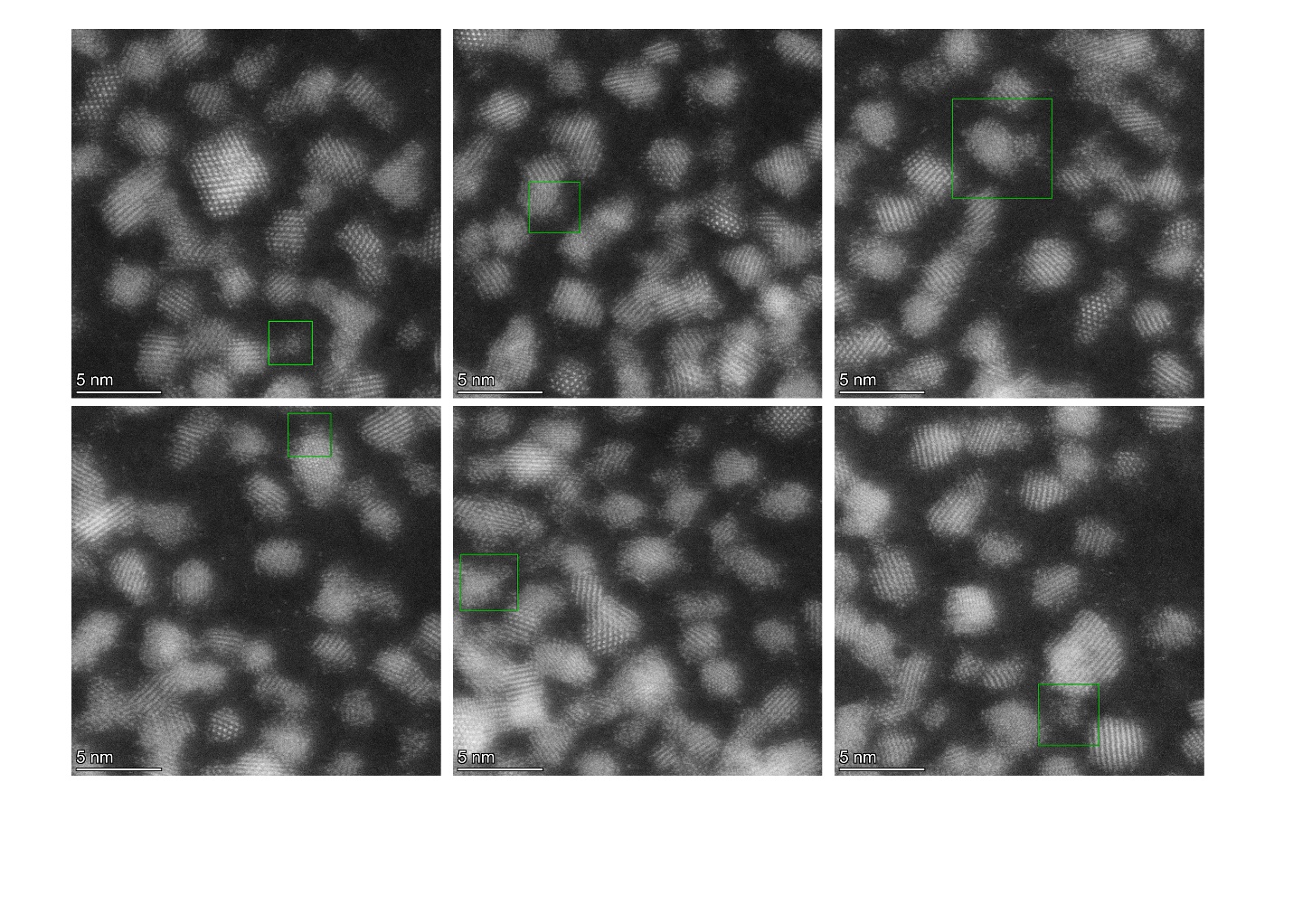


**Figure S3. HAADF-STEM image of synthesized TPP-Au_11_-CeNPs.** Green rectangles indicate the suspected Au_11_ clusters attached to CeNPs.


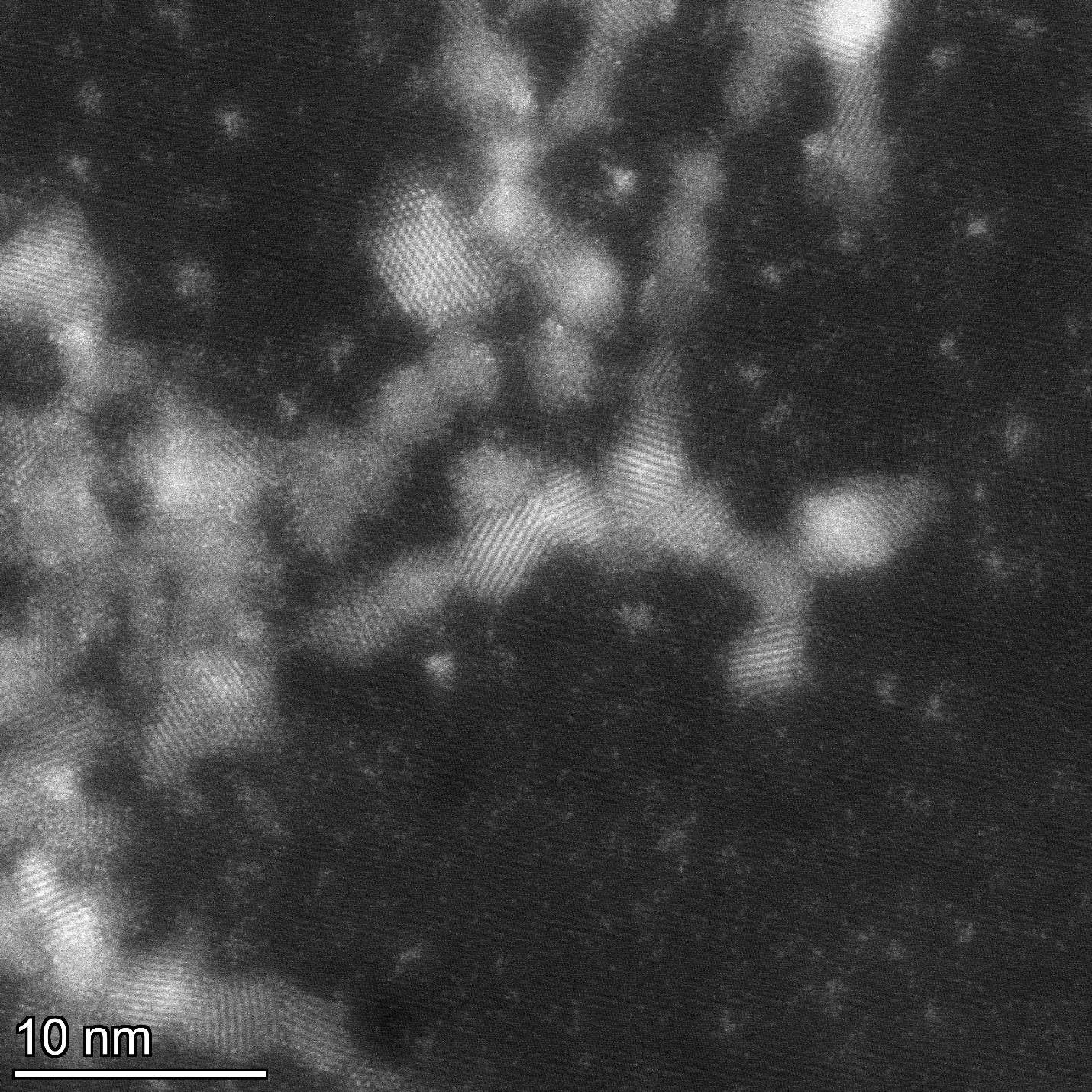


**Figure S4. HAADF-STEM image of synthesized TPP-Au_11_ nanoclusters in high concentration mixed with CeNPs.** On the image examples of Au_11_ clusters are indicated by green circles. Some clusters were overlaid with CeNPs and some were not.

**
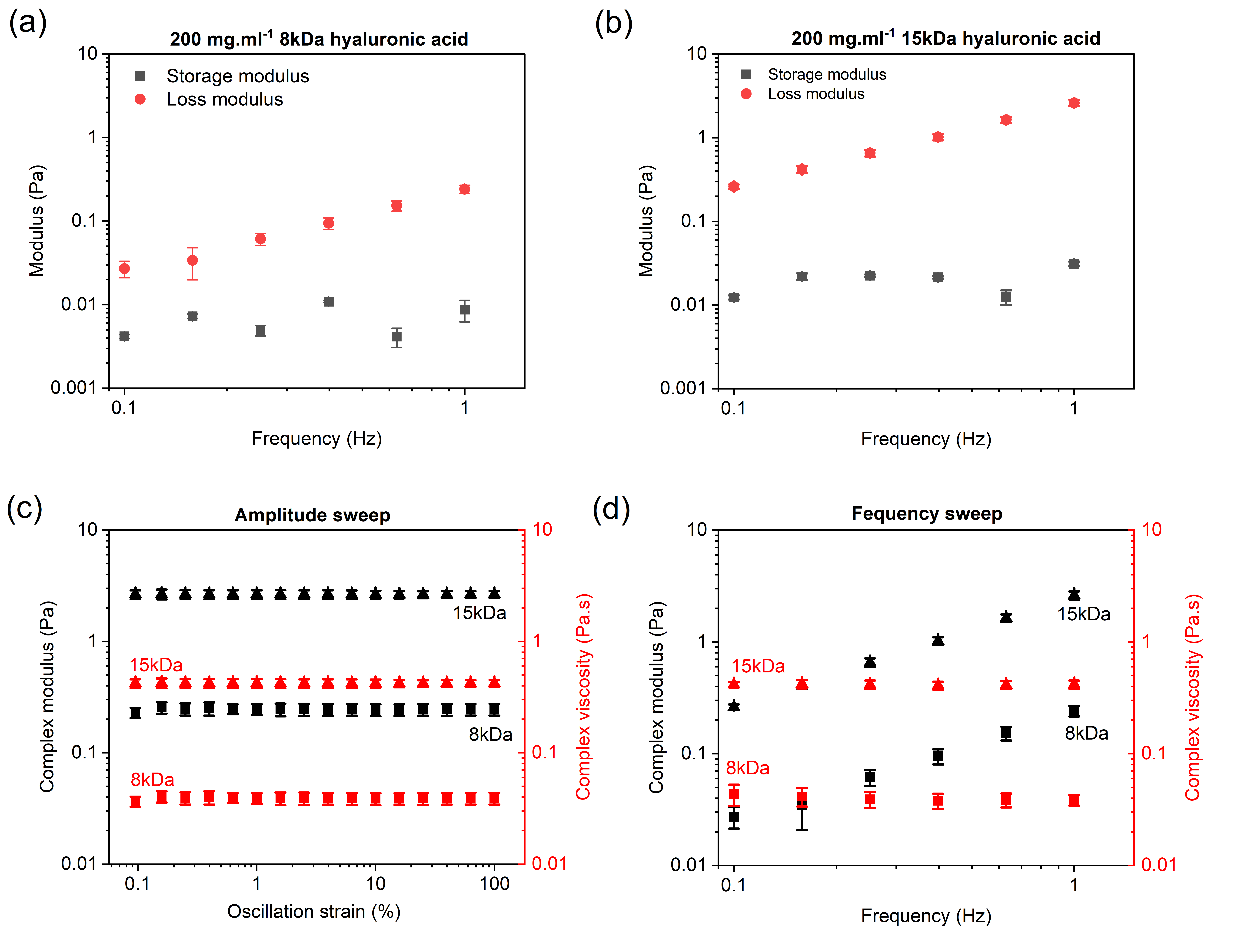
**

**Figure S5. Rheological characterization of hyaluronic acid solutions_._ (a)** Frequency sweep of the storage and loss modulus of 200 mg.ml^-1^ hyaluronic acid with a molecular weight of 8-15 kDa. **(b)** Frequency sweep of the storage and loss modulus of 200 mg.ml^-1^ hyaluronic acid with a molecular weight of 15-30 kDa. **(c)** Amplitude sweeps result in the complex modulus and viscosity of both hyaluronic acid solutions at the frequency of 1 Hz. **(c)** Frequency sweep result of the complex modulus and viscosity of both hyaluronic acid solutions at an oscillation strain of 1%.

**
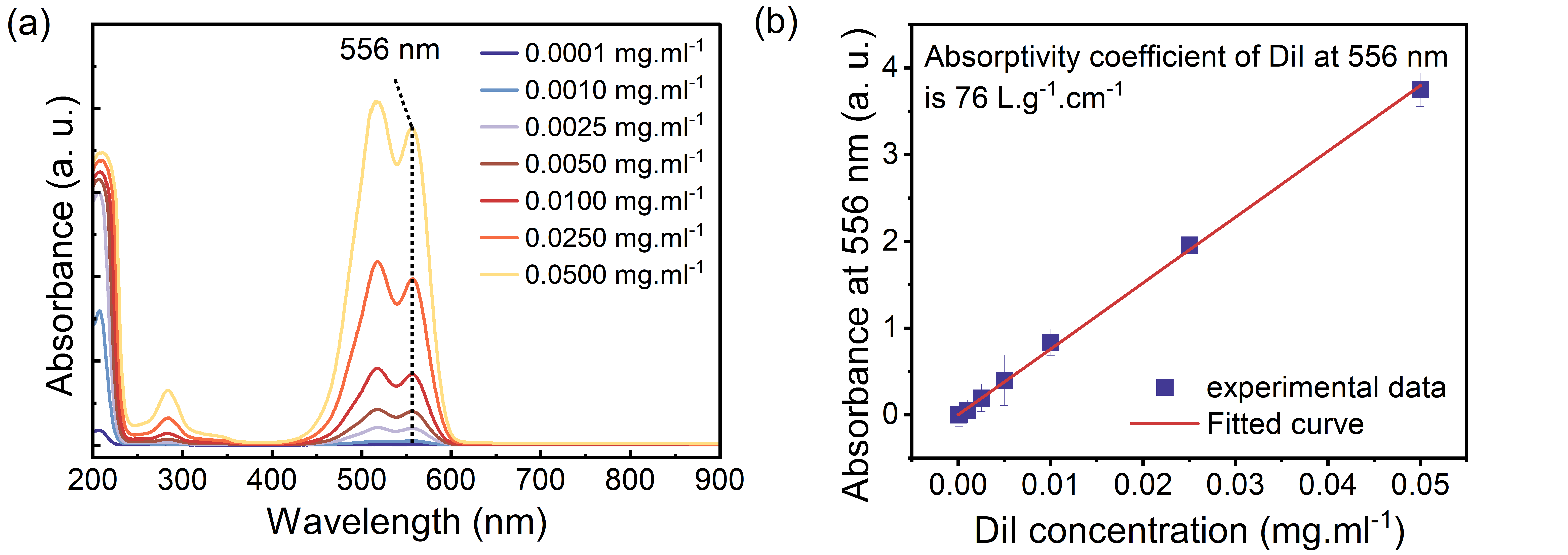
**

**Figure S6. UV-vis calibration of DiI concentration. (a)** UV-vis spectra of DiI fluorescence tags at different concentrations; and **(b)** fitted curve based on the absorbance at 556 nm. The absorptivity coefficient of DiI at 556 nm is calculated to be 76 L.g^-1^.cm^-1^ based on the Beer-Lambert Law. The coefficient is used to calculate the amount of DiI attached to the NPs.

**
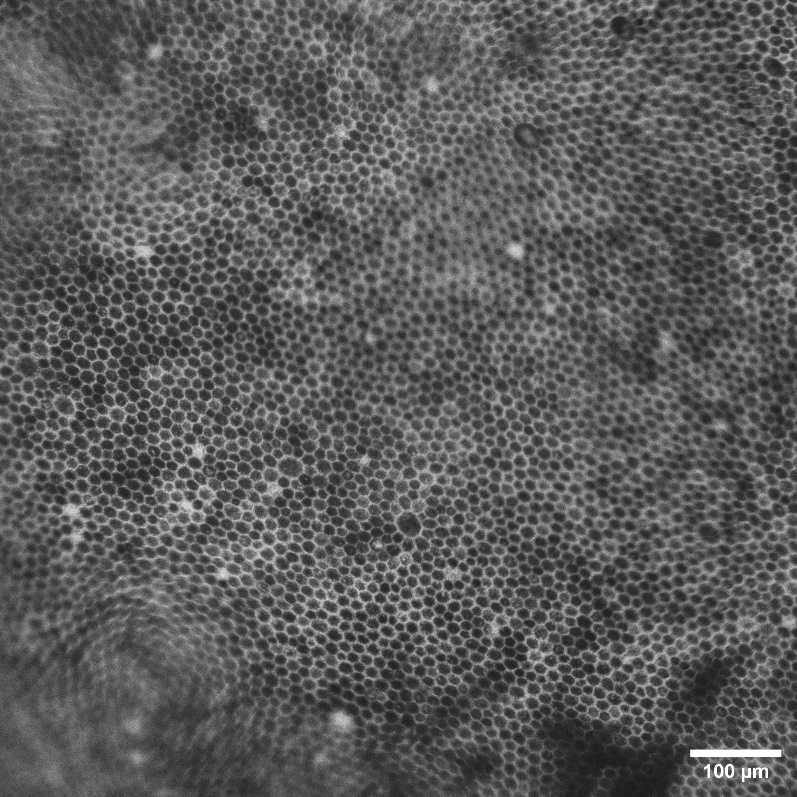
**

**Figure S7. Fluorescent image of dissected retina from resected porcine eye showing the presence of cones and rod cells.** The measured location was near the fovea. The scale bar is 100 µm.

**
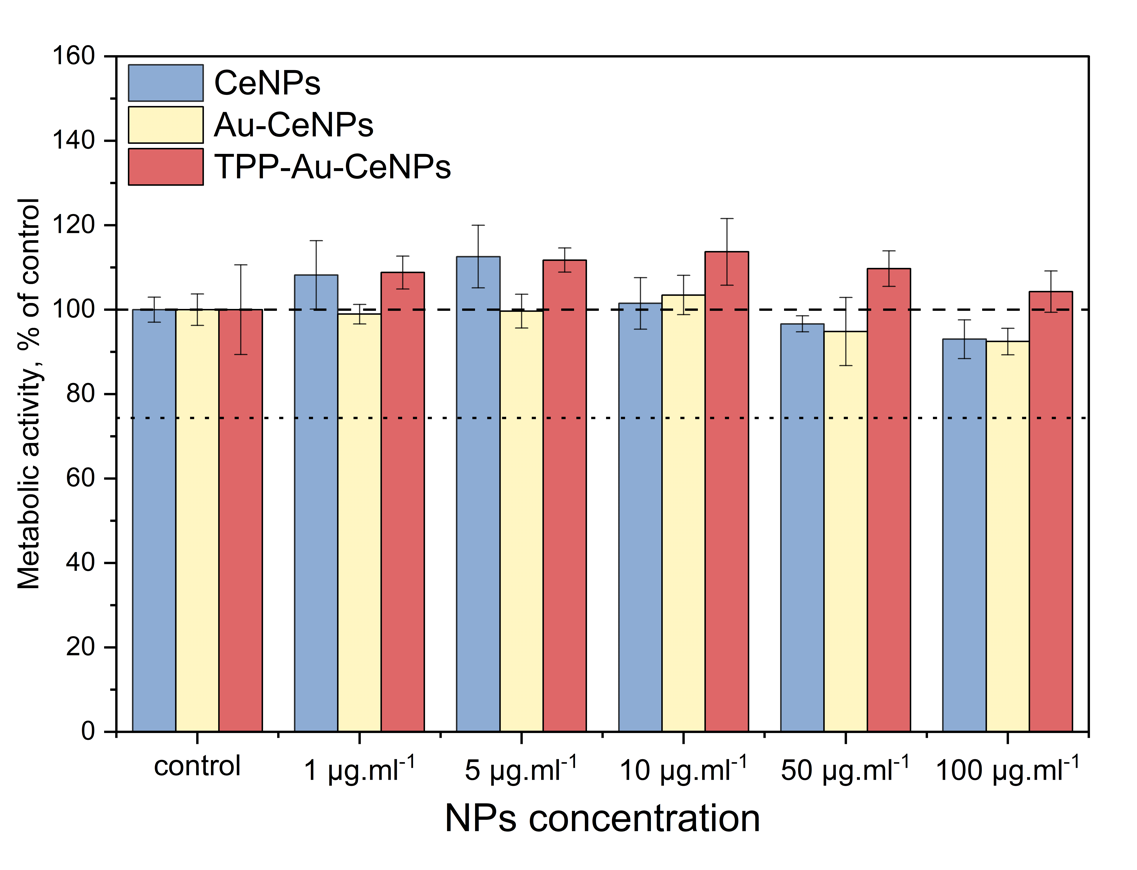
**

**Figure S8.** **Metabolic activity of HT1080 cells incubated with three types of NPs** (ranging from 1 to 100 µg.ml^-1^). The relative values of metabolic activity are expressed as a percentage of the control sample (untreated cells). The dashed line shows 100% of the control level and the dotted line shows 75% control (toxic level).


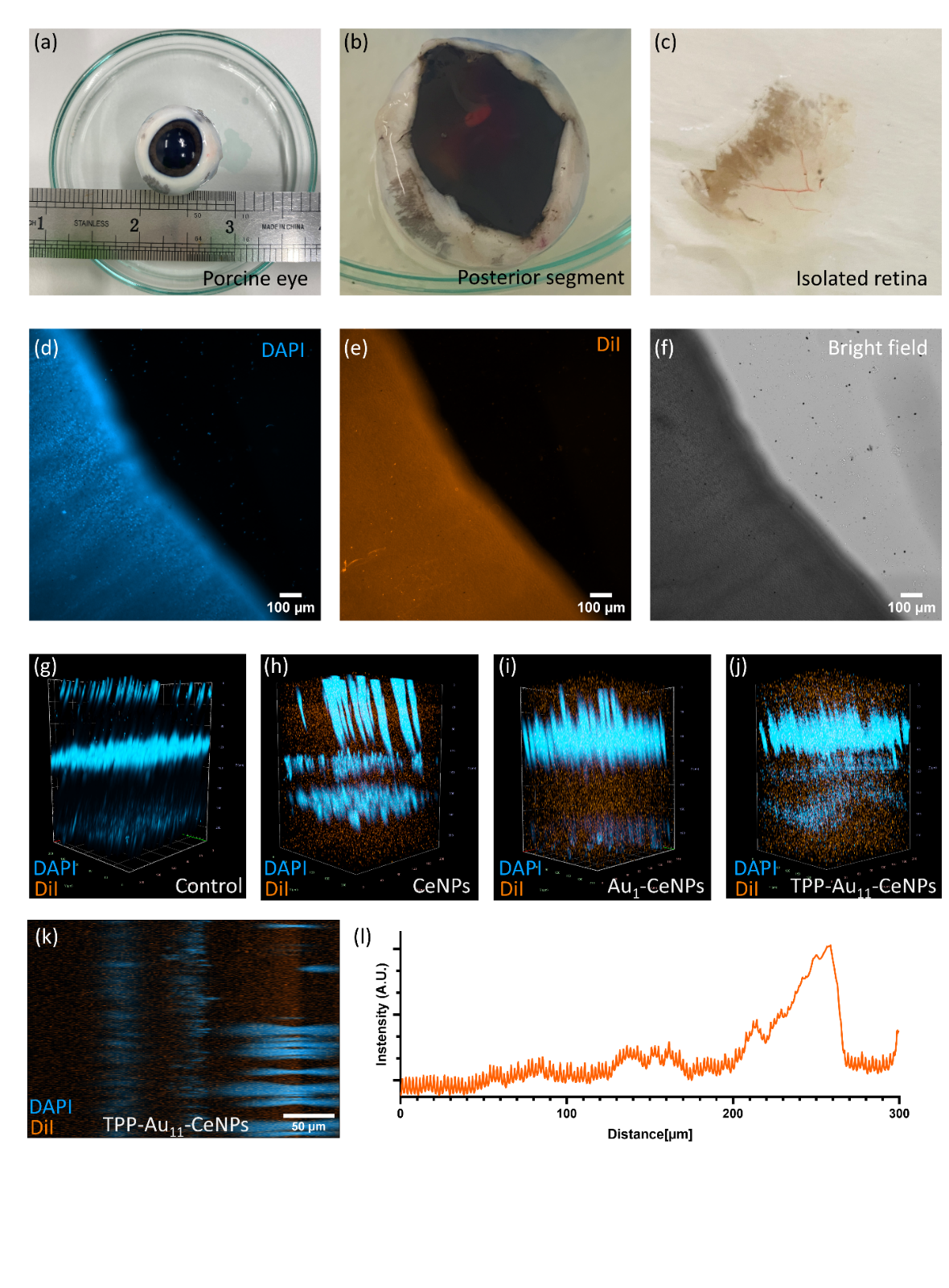
**Figure S9. Confocal imaging of nanorobots localization in retinal layer isolated from *ex vivo* porcine eyes. (a)-(c)** preparation of isolated porcine retinal layers after NPs injection. **(a)** *ex vivo* porcine eye isolated from young piglets. **(b)** posterior segment. **(c)** isolated retina. **(d)-(f)** observed fluorescence microscopic image of NP incorporated retina layers after DAPI staining, where DiI signals only came from retinal layers. **(d)** DAPI; (e) DiI tagged NPs; and **(f)** bright field. **(g)-(j)** Two-photon confocal laser scanning microscopy (CLSM) 3D projections of retinal layers after *ex vivo* intravitreal injection with three types of nanoparticles. Cell nuclei were stained with DAPI (blue), and nanoparticles were labeled with DiI (orange). **(g)** retina without particles as control; **(h)** retina with DiI-tagged CeNPs, **(i)** retina with DiI-tagged Au_1_-CeNPs, and **(j)** retina with DiI-tagged TPP-Au_11_-CeNPs. **(k)** 2D projection along the YZ plane visualizing the depth distribution of TPP-Au_11_-CeNPs within the retinal layers, and **(l)** corresponding line profile showing the DiI fluorescence intensity across the X direction.


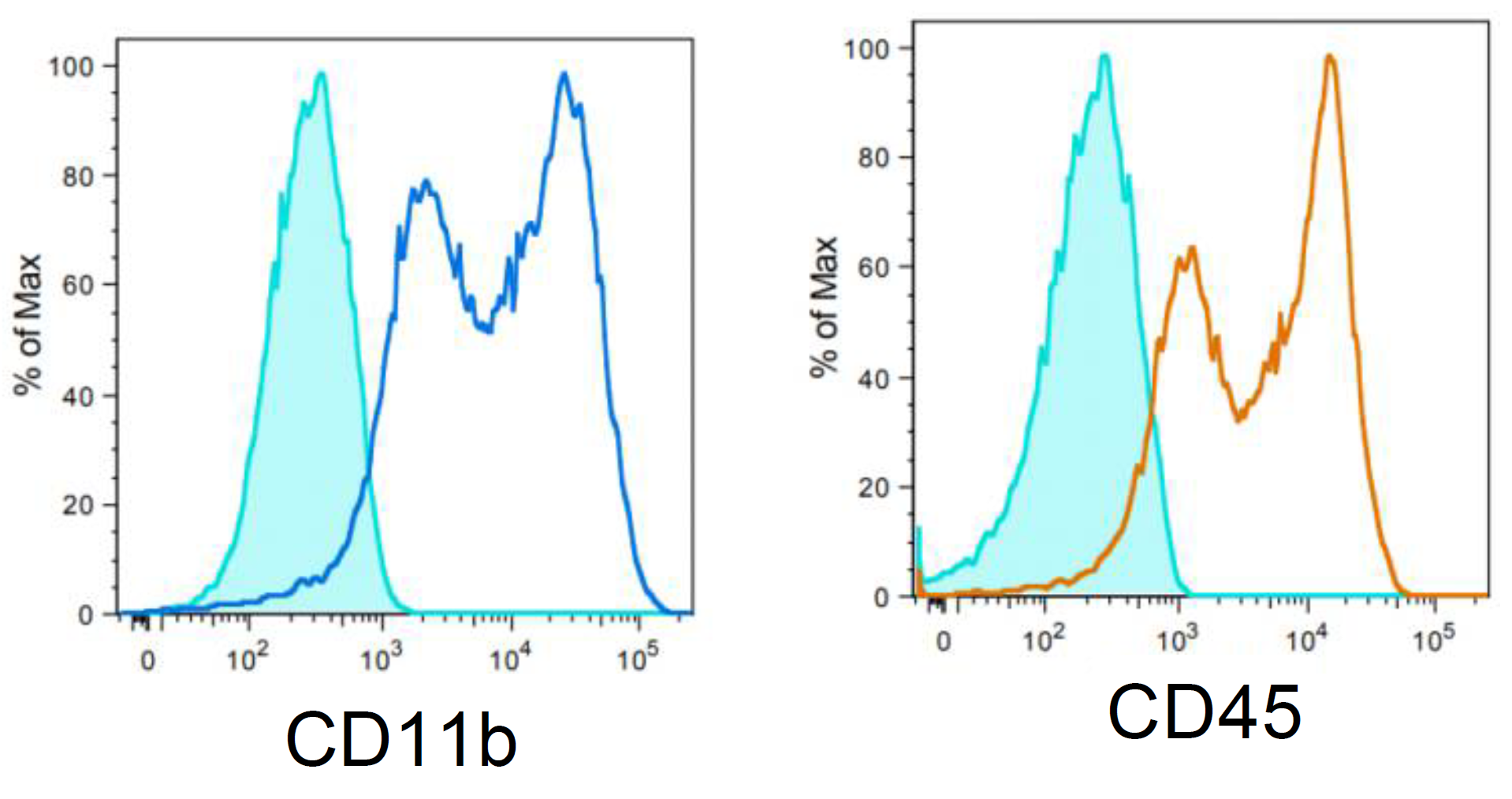


CD11b^+^

CD45^+^

(a)

(b)

**Figure S10.** Representative histograms showing the population of **(a)** CD11b^+^ and **(b)** CD45^+^ cells analyzed by flow cytometry. One example of three similar experiments is shown.

**Table S1. Sequences of Oligonucleotides used in RT-PCR**

| **Gene** | **Forward primer** | **Reverse primer** |
| --- | --- | --- |
| *Bax* | GGTCCCGAAGTAGGAGAGGA | GTGAGCGGCTGCTTGTCT |
| *Catalase* | CCCTCGGACTTTGGCAAA | CCAGACTCGAGTATCGCTGACA |
| *Galectin-3* | TGCGTTGGGTTTCACTGTGCC | GGTGCCCTATGACCTGCCCT |
| *GAPDH* | AGAACATCATCCCTGCATCC | ACATTGGGGGTAGGAACAC |
| *Gfap* | TCGAGATCGCCACCTACAG | GTCTGTACAGGAATGGTGATGC |
| *Iba-1* | CAGCATTCGCTTCAAGGACATA | ATCAACAAGCAATTCCTCGATGA |
| *IL-1β* | AGCTGGATGCTCTCATCAGG | AGTTGACGGACCCCAAAAG |
| *iNOS* | TCATTGTACTCTGAGGGCTGAC | CTTTGCCACGGACGAGAC |
| *Rhodopsin* | TGCCCTCAGGGATGTACC | ACCTGGATCATGGCGTTG |
| *Superoxide dismutase-1* | ACTTCGAGCAGAAGGCAAGC | TTAGAGTGAGGATTAAAATGAGGTC |
| *TNF-α* | GCTCCAGTGAATTCGGAAAG | GATTATGGCTCAGGGTCCAA |
| *VEGF* | TTTCTCCGCTCTGAACAAGG | AAAAACGAAAGCGCAAGAAA |

**Table S2. Antibodies for flow cytometry**

| **Antibody** | **Fluorochrome** | **Clone** | **Manufacturer** |
| --- | --- | --- | --- |
| anti-CD11b | APC | M1/70 | BioLegend |
| anti-CD11b | FITC | M1/70 | BioLegend |
| anti-CD45 | FITC | 30-F11 | BioLegend |
| anti-CX3CR1 | APC | SA011F11 | BioLegend |
| anti-MHC II | FITC | NIMR-4 | eBioscience |
| anti-IL-1β | APC | NJTEN-3 | eBioscience |

**REFERENCES**

(1) McKenzie, L. C.; Zaikova, T. O.; Hutchison, J. E. Structurally Similar Triphenylphosphine-Stabilized Undecagolds, Au_11_(PPh_3_)_7_Cl_3_ and [Au_11_(PPh_3_)_8_Cl_2_]Cl, Exhibit Distinct Ligand Exchange Pathways with Glutathione. *Journal of the American Chemical Society* **2014**, *136* (38), 13426-13435.

(2) Truttmann, V.; Drexler, H.; Stöger-Pollach, M.; Kawawaki, T.; Negishi, Y.; Barrabés, N.; Rupprechter, G. CeO_2_ supported gold nanocluster catalysts for CO oxidation: surface evolution influenced by the ligand shell. *ChemCatChem* **2022**, *14* (14), e202200322.

(3) Peters, S.; Peredkov, S.; Neeb, M.; Eberhardt, W.; Al-Hada, M. Size-dependent XPS spectra of small supported Au-clusters. *Surface Science* **2013**, *608*, 129-134.
